# Concordance in wetland physicochemical conditions, vegetation, and surrounding land cover is robust to data extraction approach

**DOI:** 10.1371/journal.pone.0216343

**Published:** 2019-05-31

**Authors:** Adam J. Kraft, Derek T. Robinson, Ian S. Evans, Rebecca C. Rooney

**Affiliations:** 1 Department of Biology, University of Waterloo, Waterloo, Ontario, Canada; 2 Department of Geography and Environmental Management, University of Waterloo, Waterloo, Ontario, Canada; University of Central Florida, UNITED STATES

## Abstract

Concordance among wetland physicochemical conditions, vegetation, and surrounding land cover may result from the influence of land cover on the sources of plant propagules, on physicochemical conditions, and their subsequent determination of growing conditions. Alternatively, concordance may result if differences in climate, soils, and species pools are spatially confounded with differences in human population density and land conversion. Further, we expect that land cover within catchment boundaries will be more predictive than land cover in symmetrical buffers if runoff is a major pathway. We measured concordance between land cover, wetland vegetation and physicochemical conditions in 48 prairie pothole wetlands, controlling for inter-wetland distance. We contrasted land-cover data collected over a four-year period by multiple extraction approaches including topographically-delineated catchments and nested 30 m to 5,000 m radius buffers. After factoring out inter-wetland distance, physiochemical conditions were significantly concordant with land cover. Vegetation was not significantly concordant with land cover, though it was strongly and significantly concordant with physicochemical conditions. More, concordance was as strong when land cover was extracted from buffers <500 m in radius as from catchments, indicating the mechanism responsible is not topographically constrained. We conclude that local landscape structure does not directly influence wetland vegetation composition, but rather that vegetation depends on 1) physicochemical conditions in the wetland that are affected by surrounding land cover and on 2) regional factors such as the vegetation species pool and geographic gradients in climate, soil type, and land use.

## Introduction

The land cover surrounding a wetland can affect the wetland and its biota by limiting species dispersal [1] or by facilitating the spread of invasive species [2] or predators [3]. Through regulation of propagule composition, abundance, and dispersal into the wetland, adjacent land covers can exhibit strong controls on wetland plant communities resulting in changes to community composition or structure [4–5]. The composition of surrounding land cover may also affect wetland biota by altering the growing conditions within the wetland. For example, the type of land cover within a wetland’s catchment may alter the volumes of snowdrift and snowmelt that enter the wetland [6]; modify evapotranspiration rates e.g., [7]; or affect the transport and accumulation of sediments [8–9], nutrients [10], salts [11] and contaminants [12]. As aquatic plant communities are structured by local environmental conditions e.g., [13–15] which “filter” the local species pool sensu [16], land cover-driven changes in wetland physicochemical conditions likely influence wetland species composition and function e.g., [11]

Regional biogeographical processes, local landscape composition and *in situ* physicochemical conditions and biotic interactions may all serve as important controls on community assembly [17]. However, these factors operate at different spatial scales, and their relative influence on community composition varies according to the region, ecosystem and taxa under consideration e.g., [18]. As such, the scale of analysis may critically affect the identification of which mechanism (regional controls such as species pool membership and climate, local controls such as the adjacent land cover types, or *in situ* controls such as water chemistry or ponded-water duration) is a stronger determinant of vegetation community composition, and even whether the mechanism is detectable [19]. Distinguishing between the effects of regional and local processes can be further complicated by heterogeneous landscapes, where changes in land cover may interact with or supersede the aforementioned processes to structure wetland communities [1]. Identifying the spatial scale (i.e. scale of effect) at which these processes are occurring is thus a necessary step in elucidating the local- and regional-level controls on community composition.

**Fig 1:**
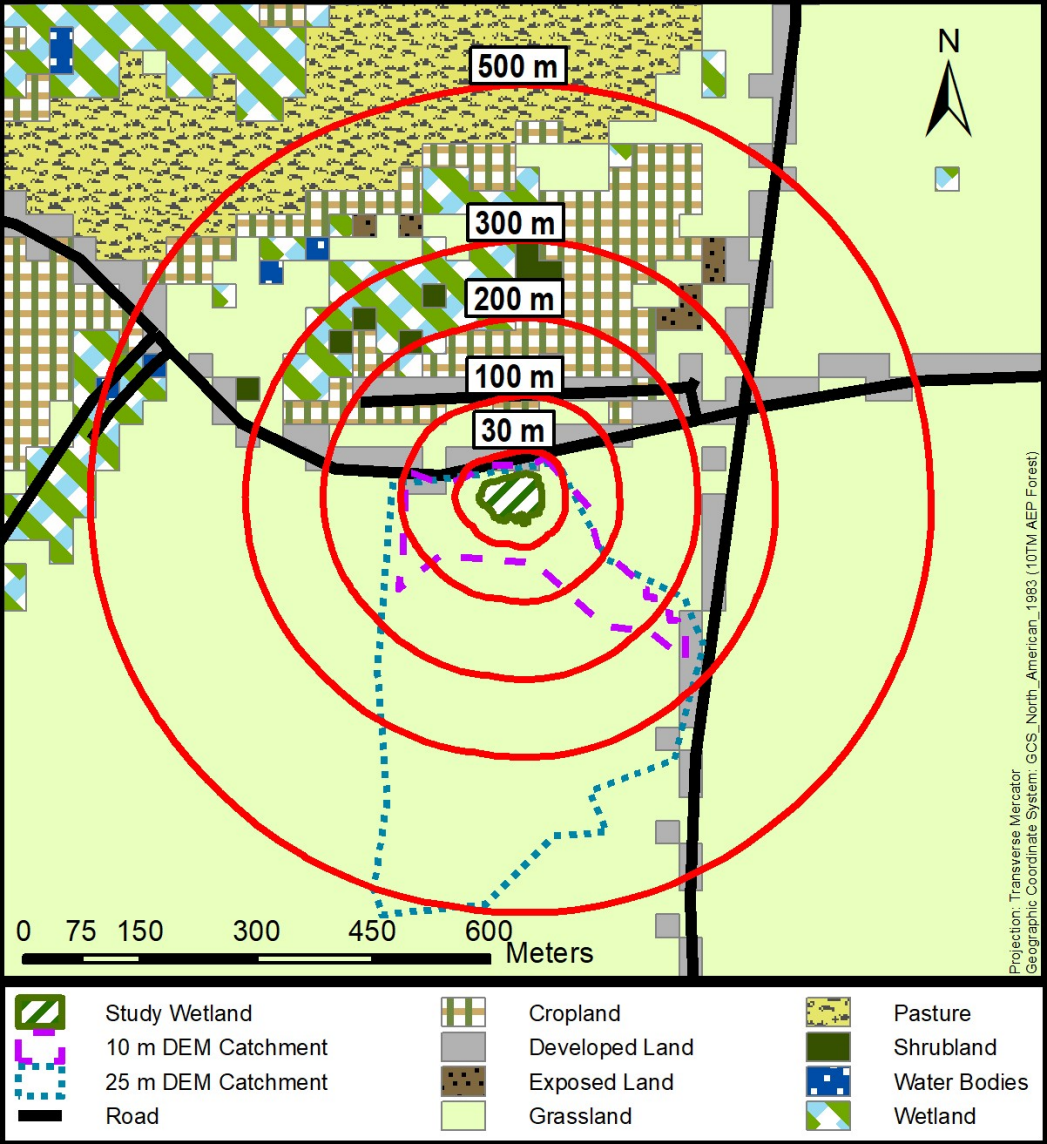
Different spatial extents used to characterize land-cover composition around study wetlands. Depicted is a 0.37 ha wetland situated in the Grassland natural region of Alberta, with land cover from 2014 (30 m pixels).

At the local scale, the spatial extent and symmetry of boundaries within which surrounding land cover influences wetland conditions remains in contention [19], with most studies using nested and symmetrical buffers to identify the scale of effect e.g.,[20–23] despite recognition of the importance of surface runoff processes in wetlands e.g., [24–25]. A major criticism of the nested and symmetrical buffer approach is that the choice of buffer radius is typically arbitrary, with little ecological relevance [19]. Further, the buffer approach presumes that there exists a discrete and critical distance at which land cover and land use affect wetland conditions that is universal for all wetlands in a specific study e.g., [23], ignoring differences in catchment size. Moreover, the approach presumes that the impact of land cover and land use is isotropic (i.e., the same in all directions) for all study sites. Yet, wetlands sit at the bottom of their catchments, not the geometric centre (Fig 1). Given that the spatial influence of runoff is anisotropic, the influence of surrounding land cover and land use on local physicochemical conditions within wetlands is also likely anisotropic.

A benefit of using symmetrical buffers for empirical analysis is that buffers around wetlands can be easily generated and visualized using a geographic information system (GIS). In contrast, delineating wetland catchments is challenging [26–27]. This is especially true in areas where low relief topography can hinder accurate delineation of catchments using digital elevation models (DEMs) [28]. In such areas, the resolution of the DEM used in catchment delineation has an influence on the catchment size and shape (e.g., Fig 1), with higher resolution DEMs typically yielding smaller catchments. Yet, it is not clear whether high-resolution DEMs are necessary or beneficial in interpreting or predicting ecological patterns. For example, higher resolution DEMs have been shown to have little effect on watershed scale simulation modeling for streams [29]. Additionally, high-resolution DEMs tend to represent barriers to water flow (e.g., roads) which may not be present on lower-resolution DEMs, further complicating catchment delineation.

We test the hypothesis that land-cover composition in the surrounding landscape influences both the physicochemical conditions and vegetation composition of prairie pothole wetlands. We explore the concordance between land cover and *in situ* wetland conditions and vegetation independent of the relative locations of those wetlands within the larger region by working in two adjacent natural regions (the Parkland and the Grassland) and by controlling for inter-wetland geographic distance in our analyses. Significant concordance between land cover and physicochemical conditions within a wetland, even after controlling for inter-wetland distances, would support our hypothesis that wetlands are affected by their surrounding landscape via the direct effect of local land covers on hydrology, chemistry, and habitat connectivity. However, understanding the sensitivity of these relationships to landscape delineation method, landscape extent, and data resolution are necessary conditions for advancing the study of wetland-landscape interactions. To help us detect any influence of temporal lag, we also test the hypothesis that land cover has an anisotropic zone of influence on wetlands driven by their catchment boundaries, using data extracted from a time series of land cover. We undertake this test by contrasting the magnitude of concordance between land cover and measures of physicochemical conditions or wetland vegetation when land cover is defined by nested and symmetrical buffers vs. when land cover is defined by topographically-delineated catchments. Finally, to determine whether wetland communities are more strongly regulated by *in situ* physicochemical conditions than by local landscape conditions (i.e. adjacent land cover) or regional factors (i.e., inter-wetland distance), we also evaluated the strength of concordance between a broad suite of physicochemical conditions and the vegetation community composition at each wetland.

## Methods

### Study region and sites

**Fig 2:**
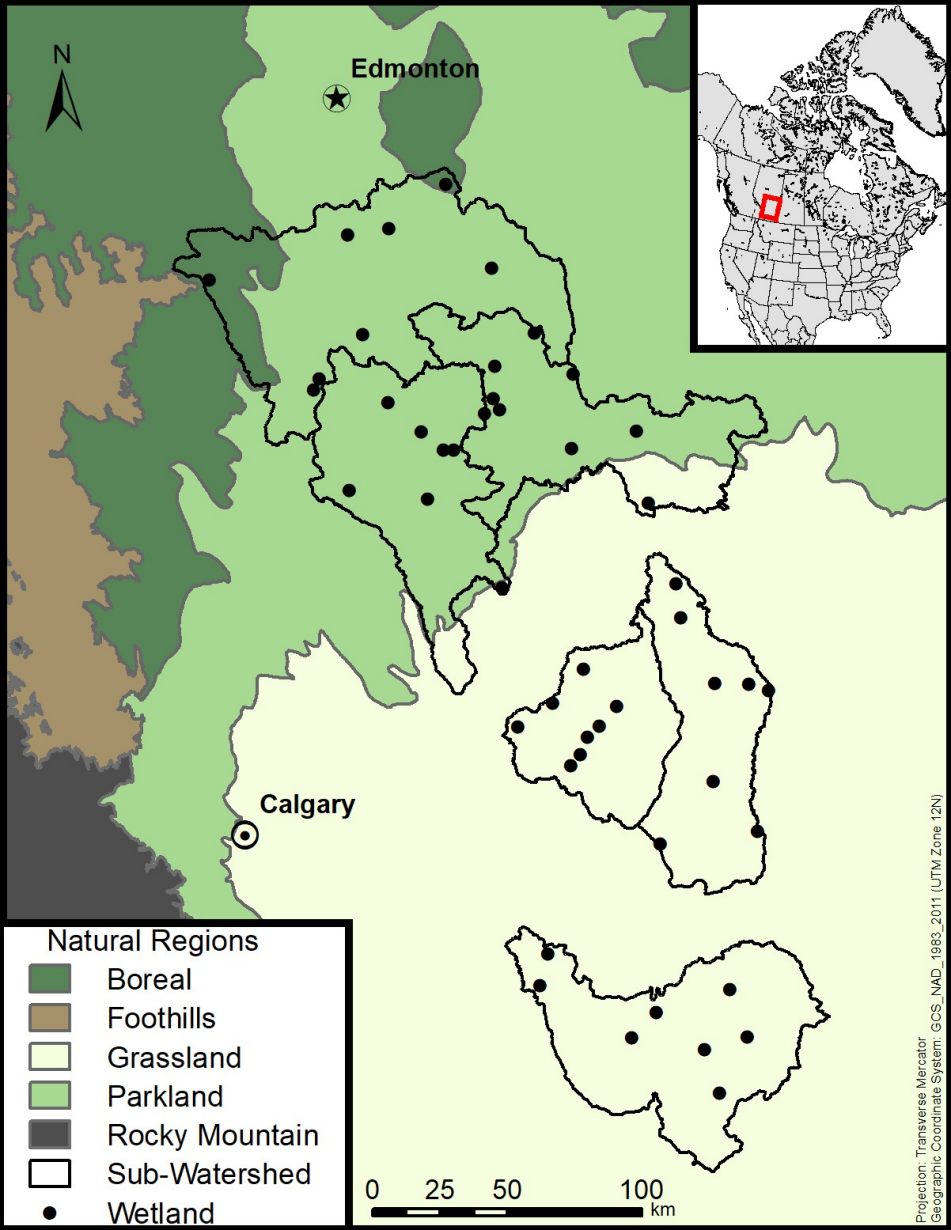
Distribution of wetlands sampled in 2014 (*n* = 48) within six major sub-watersheds in the Grassland and Parkland natural regions of southern Alberta, Canada.

We sampled the Grassland and Parkland natural regions of Alberta, Canada, within the glaciated plains (“Prairie Pothole Region”) of North America (Fig 2). Both regions have undulating topography, with poorly-drained soils comprising clay-rich glacial tills [25]. Graminoid marshes form in the resulting topographic depressions (prairie potholes). Groundwater recharge occurs at some wetlands, but most infiltrated water is retained in upland soils [25]. These prairie pothole marshes receive most of their water from snowmelt runoff since the semi-arid climate creates a moisture deficit the rest of the year (i.e., potential evapotranspiration typically exceeds precipitation), and thus summertime runoff is very low [25]. The pattern of potholes combined with temperature and precipitation regimes limit surface-water connectivity to infrequent summer deluge conditions, in which a “fill-spill” effect may occur between adjacent basins [24]. Due in part to the low surface-water connectivity among wetlands and the semi-arid climate, wetland water levels draw down gradually over the growing season through evapotranspiration and soil infiltration. Many prairie pothole wetlands dry out completely every summer, producing a seasonal cycle of spring fill and summer draw down. The periodicity of ponding serves as the basis of a classification system that categorizes the prairie pothole wetlands as ephemeral, temporary, seasonal, semi-permanent, or permanent, reflecting the duration of ponded water [30].

**Fig 3:**
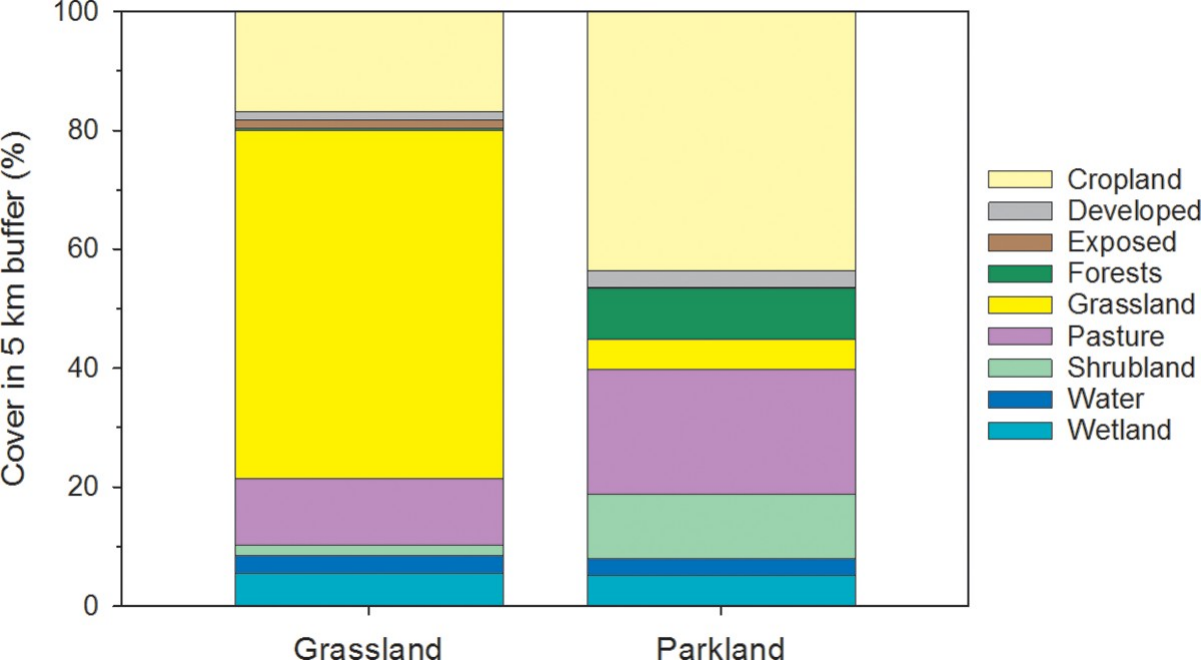
Comparison of study wetlands in the Grassland and Parkland natural regions. Values represent the average composition of each of 9 land-cover classes surrounding wetlands in the Grassland (*n* = 24) and Parkland (*n* = 24) measured within a 5 km symmetrical buffer.

Climatic conditions differ between the more northern Parkland (mean annual temperature 2.3°C, precipitation 441 mm) and southern Grassland (mean annual temperature 4.2°C, precipitation 371 mm) [31], supporting different natural and anthropogenic land covers in each region (Fig 3); S1 Table). Agriculture is a dominant land use in both natural regions: primarily cereal and oilseed cultivation in the Parkland, and a mix of irrigated cropping and rangeland for cattle in the Grassland. Forests and shrublands are more prevalent in the Parkland, whereas these habitats are scarce in the Grassland. Instead, the majority of the Grassland natural region is occupied by native grassland (albeit subject to grazing pressures). Urban areas and oil and gas exploration are also present in both natural regions. The land cover immediately surrounding a wetland can range from being entirely natural (e.g., native grassland or forest) to some adjacent anthropogenic cover (e.g., a road) to anthropogenic land cover entirely surrounding (and possibly occupying part of) a wetland (e.g., cropping, grazing).

**Fig 4:**
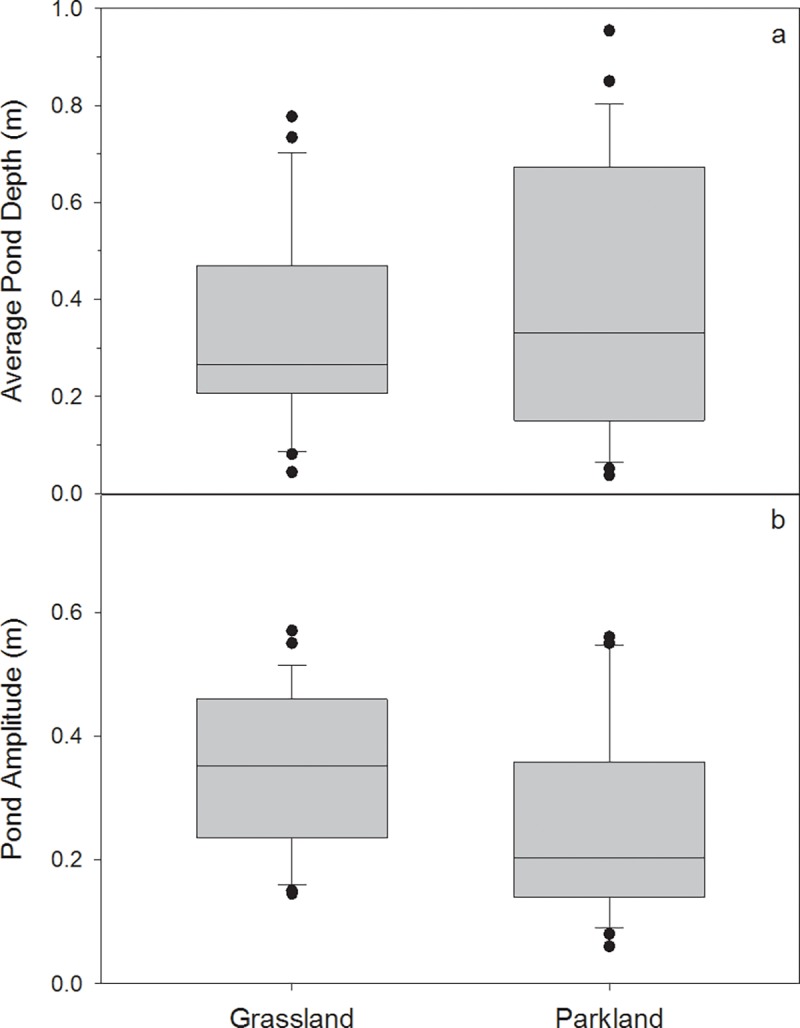
Comparison of a) average pond maximum depth measured in May 2014; and b) pond seasonal amplitude (i.e., seasonal maximum depth minus seasonal minimum depth, measured at the wetland’s deepest point) of wetlands in the Grassland (*n* = 24) and Parkland (*n* = 24) natural regions.

**Fig 5:**
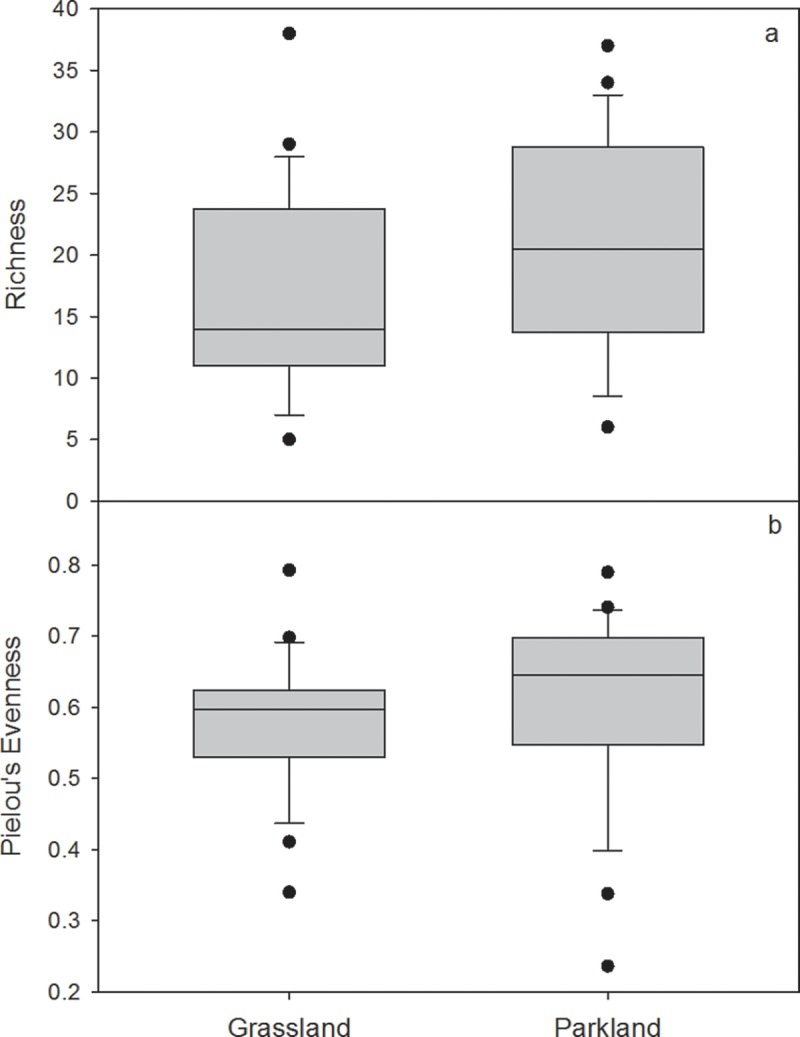
Comparison of a) species richness; and b) evenness of vegetation in wetlands in the Grassland (*n* = 24) and Parkland (*n* = 24) natural regions.

The differences in climatic conditions between the Parkland and Grassland result in differences in wetland vegetation communities and physical condition (S1 File). The cooler, wetter climate of the Parkland results in more permanently-ponded wetlands than in the Grassland (Fig 4), though a range of permanence classes exist in both natural regions. Similarly, the regional species pool differs between the Parkland and Grassland natural regions. Parkland wetlands tend to support more willow shrubs (*Salix* spp.) and sedges like water sedge (*Carex aquatilis*) or wheat sedge (*Carex atherodes*), whereas the drier Grassland wetlands are typically dominated by grasses like sloughgrass (*Beckmannia syzigachne*), foxtail barley (*Hordeum jubatum*) and fowl bluegrass (*Poa palustris*) (S1 File). Vegetation in Parkland wetlands is on average slightly more diverse than in the Grassland (Fig 5), possibly because climate conditions in the Parkland support more woody vegetation. Differences in the dominant anthropogenic land uses–cultivation vs. pasture–may also modify the regional species pools [18] and affect wetland community composition [2]. Despite these differences in species composition, wetland vegetation zonation in both natural regions generally reflects typical prairie pothole configurations, where zones are distributed concentrically within the wetland basin and are strongly tied to the permanence of ponded water [30].

### Site selection

We selected three sub-watersheds from each natural region that were mainly composed of glaciolacustrine or glaciofluvial landforms. Within each sub-watershed we compiled a random sample of all temporarily-ponded to semi-permanently-ponded marshes from the Alberta Merged Wetland Inventory [32] such that our sample frame was stratified along two independent gradients. The first gradient was a range of permanence class and the second was a gradient of human disturbance, which we measured as the proportion of non-natural land cover (i.e., developed, cropland, pasture) within a 500 m buffer surrounding each wetland during 2013 [33]. Selection of the final sampling sites was subject to property access and ground-level verification of the wetland permanence class and disturbance levels. We maintained a distance of at least 3.5 km between wetlands to ensure their spatial independence. Based on these criteria, we selected eight marshes within each sub-watershed for a total of 48 sites within the study region (geographic distribution of the wetlands ranged from 50.16°N to 53.23°N latitude and from 111.22°W to 114.20°W longitude; Fig 2). Sites were visited five times between May and August 2014 in a stratified random order to prevent sample date confounding with disturbance level or permanence class and to ensure that sites were not repeatedly sampled at the same time of day, as this may influence physicochemical conditions. Sampling permission for all sites on private land was granted by the landowners, and sampling in provincially-protected areas was authorized by Alberta Tourism, Recreation and Parks under research permits 14–049 and 14–075.

### Physicochemical measurements

Given a lack of certainty about the relationship between physicochemical variables and wetland vegetation, we adopted an inductive approach by recording measurements for a large number of commonly cited variables (*n =* 45, see comprehensive list in S2 Table). These included hydrologic, water quality and sediment quality measures often cited as important determinants of wetland vegetation, such as water depth and drawdown [34], nutrient levels [35], salinity [36], clarity [37], and common agrochemical contaminants.

Detailed water chemistry analysis methods are available in [38]. On each of the five site visits, we monitored water depth with staff gauges and measured *in situ* turbidity (AquaFluor, Turner Designs), pH (IQ150, Spectrum Technologies), dissolved oxygen (DO; HQd Portable Meter and LDO101, Hach Company), conductivity and temperature (HQd Portable Meter and CDC401, Hach Company). We collected water samples in May for pesticide analysis. Neonicotinoids (thiamethoxam, clothianidin, and imidacloprid), glyphosate and residues of its derivatives, aminomethylphosphonic acid and glufosinate were measured by the Alberta Innovates Environmental Analytical Services Laboratory (Vegreville, AB). An additional 104 commonly detected pesticides were measured at the Agriculture and Agri-Food Canada Lethbridge Research Centre (Lethbridge, AB), including 2,4-D, difenoconazole and MCPA. We also collected a water sample for analysis of nutrients (total nitrogen and total phosphorus), major ions (Ca^2+^, Mg^2+^, K^+^, Na^+^, Cl^-^ and SO_4_^2-^), total suspended solids, total carbon and dissolved organic carbon by the University of Alberta Biogeochemical Analytical Services Laboratory (Edmonton, AB).

Wetland soil sampling took place during peak plant biomass (July-August). *In situ* measurements of soil conductivity (HI98331, Hanna Instruments) were taken at all vegetation quadrats (see “Vegetation sampling”, below). At three quadrats per vegetation assemblage, we used a suction corer of 4.9 cm inner diameter to extract three replicate soil cores to a depth of 10 cm. These cores were composited and analysed for bulk density and water content gravimetrically after drying soil at 80°C for 72 h.

Loss-on-ignition was determined following 4 h in a muffle furnace at 550°C. Soil was also analyzed for total carbon and nitrogen at the University of Alberta Biogeochemical Analytical Services Laboratory (Edmonton, AB). Additional soil fertility analysis (including measurement of soil pH (saturation paste), K^+^, Na^+^, Mg^2+^, Ca^2+^, Zn, Mn and S) was conducted at the University of Guelph Agriculture and Food Laboratory (Guelph, ON). Lastly, extraction and analysis of soil pesticide residues was performed at the Agriculture Agri-Food Canada-Lethbridge Research Centre (Lethbridge, AB), including the same suite of 104 commonly detected pesticide compounds.

### Vegetation sampling

Vegetation sampling occurred from mid-July to mid-August 2014, when most plants were at peak aboveground biomass. We mapped the wetland-upland boundary using a handheld GPS receiver with 1.86 m horizontal accuracy (Juno Trimble T41/5 running ArcPad v. 10.0 [39] and SXBlue II GNSS Receiver) according to the distribution of wetland obligate and facultative wetland plants. Vegetation assemblages were identified and delineated according to the combination of vegetation form (e.g., forb, robust emergent, shrub) and identity of dominant or co-dominant species (>25% cover) within a patch, following an established wetland vegetation mapping protocol [40].

In each wetland, we deployed a minimum of five 1 m^2^ quadrats randomly within each vegetation assemblage that exceeded an area of 100 m^2^, such that our sampling intensity reflected the complexity and relative composition of the wetland vegetation assemblages. Within each quadrat, vascular plants were identified to the species-level where possible, following [41], and the percent cover of each species was recorded (+/- 5% cover). Plant abundance data were updated to reflect the nomenclature and current taxonomic status accepted by the Integrated Taxonomic Information System (http://www.itis.gov/; accessed January 2016).

### Spatial analyses

Land-cover data were obtained from Agriculture and Agri-Food Canada’s (AAFC) Annual Crop Inventory for 2011, 2012, 2013, and 2014 [33, 42–44], which classifies land cover in Canada’s arable regions at a 30 m spatial resolution with ≥ 85% accuracy within our study region [45]. Four years of land cover data were selected to determine whether using data that coincide with field measurements or recent historical data affects the strength of detected landscape-wetland condition concordance. Due to the high thematic resolution of the AAFC data (comprising 44 land-cover classes of which 30 are agricultural variants), we reclassified the data into the following nine land-cover classes based on ecological function or similar land use and land management activities: forests, wetlands, native grassland, shrubland, cropland, pasture/hay, developed, water bodies, or exposed/barren land.

For each of our 48 study sites, we produced eight nested buffers of varying radii (30, 100, 200, 300, 500, 1,000, 2,000, and 5,000 m) extending from each wetland’s perimeter, as defined by our vegetation mapping. In addition, we delineated wetland catchments using 10 m and 25 m resolution digital elevation models (DEMs) acquired from Alberta Innovates Technology Futures and AltaLIS, following the delineation approach of [28].

For each year, we calculated the proportional coverage of land-cover classes within each of the eight buffers and two catchments, yielding 40 different land-cover datasets. There were no significant changes in land cover among the four years when measured at the largest spatial extent, i.e., the 5,000 m buffers (one-way ANOVA: *F*_3, 188_ = 0.0499, *p* = 0.985). This eliminates the possibility of temporal lag effects due to land-cover change during our study period, though it does not suggest land management practices were constant. We proceeded using the proportional coverage of land cover, rather than absolute area, because it provided a standardized measurement across wetlands with different areal extents (Table 1). Map production and spatial analyses were performed in ArcMap, v. 10.3.1 [46].

**Table 1:** Summary statistics of the size of wetlands and the ten spatial extents used to extract land-cover data around each wetland for all study sites (*n* = 48) and for wetlands in the Grassland (*n* = 24) and Parkland (*n* = 24) natural regions separately. Units for all values are hectares.

Spatial Extent	Sites	Mean	Median	Standard Deviation	Range
Wetland	All	0.81	0.50	0.81	0.04–3.28
	Grassland	0.98	0.83	0.91	0.04–3.24
	Parkland	0.63	0.41	0.67	0.10–3.28
10 m DEM Catchment	All	17.24	12.42	16.79	2.51–76.01
	Grassland	23.40	17.31	21.38	3.37–76.01
	Parkland	11.08	11.16	6.33	2.51–25.14
25 m DEM Catchment	All	21.74	18.03	17.37	2.95–72.94
	Grassland	29.05	23.78	20.31	3.74–72.94
	Parkland	14.43	11.68	9.62	2.95–31.40
30 m Buffer	All	1.55	1.25	0.83	0.52–3.92
	Grassland	1.67	1.56	0.87	0.52–3.51
	Parkland	1.43	1.17	0.80	0.70–3.92
100 m Buffer	All	7.13	6.26	2.48	3.93–13.74
	Grassland	7.57	7.27	2.70	3.93–13.36
	Parkland	6.70	6.03	2.21	4.48–13.74
200 m Buffer	All	20.38	18.74	4.74	14.13–32.61
	Grassland	21.27	20.75	5.25	14.13–32.27
	Parkland	19.48	18.28	4.08	15.23–32.61
300 m Buffer	All	39.88	37.50	6.97	30.61–57.68
	Grassland	41.24	40.50	7.76	30.61–57.27
	Parkland	38.53	36.81	5.93	32.25–57.68
500 m Buffer	All	97.72	93.84	11.41	82.41–126.58
	Grassland	99.98	98.85	12.76	82.41–125.94
	Parkland	95.46	92.70	9.61	85.13–126.58
1,000 m Buffer	All	352.18	344.58	22.46	321.78–408.60
	Grassland	356.70	354.59	25.21	321.78–407.31
	Parkland	347.65	342.33	18.78	327.21–408.60
2,000 m Buffer	All	1332.01	1317.01	44.54	1271.47–1443.43
	Grassland	1341.06	1337.03	50.07	1271.47–1440.86
	Parkland	1322.97	1312.55	37.09	1282.32–1443.43
5,000 m Buffer	All	8039.11	8000.97	110.84	7888.01–8315.38
	Grassland	8061.80	8051.98	124.70	7888.01–8308.81
	Parkland	8016.41	7990.24	92.08	7915.06–8315.38

### Correlation between land cover and physicochemical and vegetation observations

To evaluate the concordance between land cover and wetland physicochemical or vegetation conditions, we used partial Mantel tests [47–48]. To explore the physicochemical and vegetation data visually and to inform our approach to concordance analysis, we also employed ordination methods to visually explore and summarize the vegetation and physicochemical data (S1 File).

Mantel tests evaluate the correlation (*r*_M_) between two dissimilarity matrices and describe the extent to which the two dissimilarity matrices exhibit the same pattern of redundancy. Because geographic proximity and concepts such as spatial autocorrelation among samples may artificially inflate *r*_M_ values [49], we used partial Mantel tests [48, 50] to quantify the effect of location and proximity among sites. The partial Mantel test allows us to statistically control for the linear effect of inter-wetland distances before calculating the Mantel correlation between another pair of distance matrices [49]. Mantel *r*_M_ values are usually much smaller than the Pearson’s correlation coefficients produced for the same sample size [51]: *r*_M_ coefficients ≥ 0.1 usually indicate a strong association between the two dissimilarity matrices e.g., [23, 51]. The statistical significance of the Mantel correlation (*r*_M_) is determined by repeated randomization of the rows and columns of one matrix, where the resulting *p*-value represents the proportion of randomized permutations with a correlation score larger than the observed score. However, our purposes are comparative; therefore, we are more interested in the relative magnitude of Mantel *r*_M_ values derived using different catchment or buffer sizes to extract land cover than we are in the statistical significance of individual *r*_M_ values.

Our comparison between land cover and physicochemical variables used Euclidean distances to construct the dissimilarity matrices, because these datasets exhibited bivariate linearity and low sparsity. To generate the dissimilarity matrix describing physicochemical variation among sites to use in our partial Mantel tests, we standardized the 45 physicochemical variables by relativizing each measurement by the variable’s maximum observed value [47]. Repeated *in situ* measurements were averaged across site visits to provide one value per wetland. We also consolidated pesticide data by type, yielding the incidence of herbicide, insecticide and fungicide detections in water and soil. We took the average of soil nutrients, ions and physical parameters across quadrats to obtain wetland-level soil-quality data.

In contrast, we used the Bray-Curtis distance measure [52] to calculate the dissimilarity matrices when comparing vegetation relative abundance to land cover or to physicochemical conditions, because vegetation community datasets typically possess high sparsity and because species vary naturally in their maximum abundance [47] To produce a dissimilarity matrix describing variation in vegetation community composition among sites, we used the relative cover of each plant species at a wetland, averaging across all quadrats sampled at each site. While rare species may be important from a conservation perspective, they may skew the results of concordance analysis [47]. When a plant species was observed in only one of the 150 mapped assemblages, we excluded it from the dissimilarity calculations to reduce dataset sparsity. This left 121 plant species for analysis.

Latitudinal trends in climate and land cover types associated with the natural regions spanning our study may violate the Mantel test assumptions of monotonicity and homoscedasticity between the dissimilarity matrices, resulting in a loss of statistical power [48]. To ensure that the underlying spatial structure in our data was linear and that the partial Mantel test would thus be appropriate, we first analyzed our data spatially by reducing the number of physicochemical and vegetation variables down to four principal components (PCs), each with an eigenvalue greater than one (S1 File). We identified a slight positive global spatial autocorrelation for each principal component using Moran’s I (PC1 0.17, PC2 0.26, PC3 0.19, and PC4 0.22) GIS [46]. We then fit an experimental variogram to a semivariance point cloud for each principal component using 12 lags, size 0.0634966, to 8 mathematical models (circular, spherical, tetraspherical, pentaspherical, exponential, guassian, rational quadratic, and stable) with GIS [46]. Results of the experimental variograms identified slight directionality to the spatial autocorrelation in a north-south direction with some variability by mathematical model and PC axis. The calculated range varied between 27 and 57 km, along the major axis of autocorrelation, and the farthest distance among sites was 358 km. Despite our sampling efforts to ensure spatially independent samples (obtained at a distance greater than 3.5 km as determined by spatial autocorrelation among wetland land cover data), our measurements show slight linear spatial autocorrelation along the North-South latitudinal gradient.

This is appropriate because the underlying spatial structure followed a linear North-South latitudinal gradient [49]. We compared 40 land-cover dissimilarity matrices with each of the physicochemical and vegetation dissimilarity matrices, using the inter-wetland distances as a third “control” matrix, for a total of 80 partial Mantel tests. Each partial Mantel test used 10,000 permutations of a Monte Carlo randomization test to quantify the significance of the calculated *r*_M_ value [53]. To compare the *r*_M_ values among the 40 spatial extent × year combinations, we used bootstrapping to generate 90^th^ percentile confidence intervals around each *r*_M_ value [54]. Bootstrapping involved 5,000 iterations at a resampling rate of 0.7, without replacement. A partial Mantel test was also used to test for concordance between physicochemical and vegetation dissimilarity matrices after controlling for inter-wetland distance, using the Bray-Curtis distance measure for all three dissimilarity matrices. All partial Mantel tests and bootstrapping were performed in the statistical platform *R*, v. 3.2.3 [55] using the Mantel function of the “ecodist” package [54].

## Results

### Wetland, catchment, and buffer spatial characteristics

Typical of prairie pothole wetlands, our study wetlands were generally small (Table 1). Wetland catchments delineated using DEMs of differing resolution differed in areal extent and shape: catchments delineated from the 10 m DEM were smaller than the 25 m DEM catchments (two-tailed paired sample *t*-test: *t* = -3.279, *df* = 47, *p* = 0.002) and the 10 m DEM catchments had a greater size range (Table 1). On average, catchments in the Grassland were twice as large as those in the Parkland, as measured using both DEMs, resulting in a smaller mean wetland to catchment size ratio. Regardless of DEM resolution, mean catchment areas were most similar to the areas of the 200 m buffers (two-tailed paired sample *t*-test: *t* = -1.487, *df* = 47, *p* = 0.144 for 10 m and *t* = 0.586, *df* = 47, *p* = 0.561 for 25 m DEM, indicating no significant difference in size), although catchment area was considerably more variable than area within the 200 m buffers (Table 1), indicating greater skew in the distribution of catchment sizes.

### Concordance among matrices

As the first step in the partial Mantel tests, we measured concordance between a matrix of inter-wetland distances and the physicochemical and vegetation community composition dissimilarity matrices to control for the influence of wetland location. These Mantel tests revealed strong and statistically significant concordance between wetland location and physicochemical condition (*r*_M_ = 0.191, *p* = 0.0030) and vegetation (*r*_M_ = 0.203, *p* <0.0001). Note that these are some of the largest *r*_M_ values we observed in our study.

**Fig 6:**
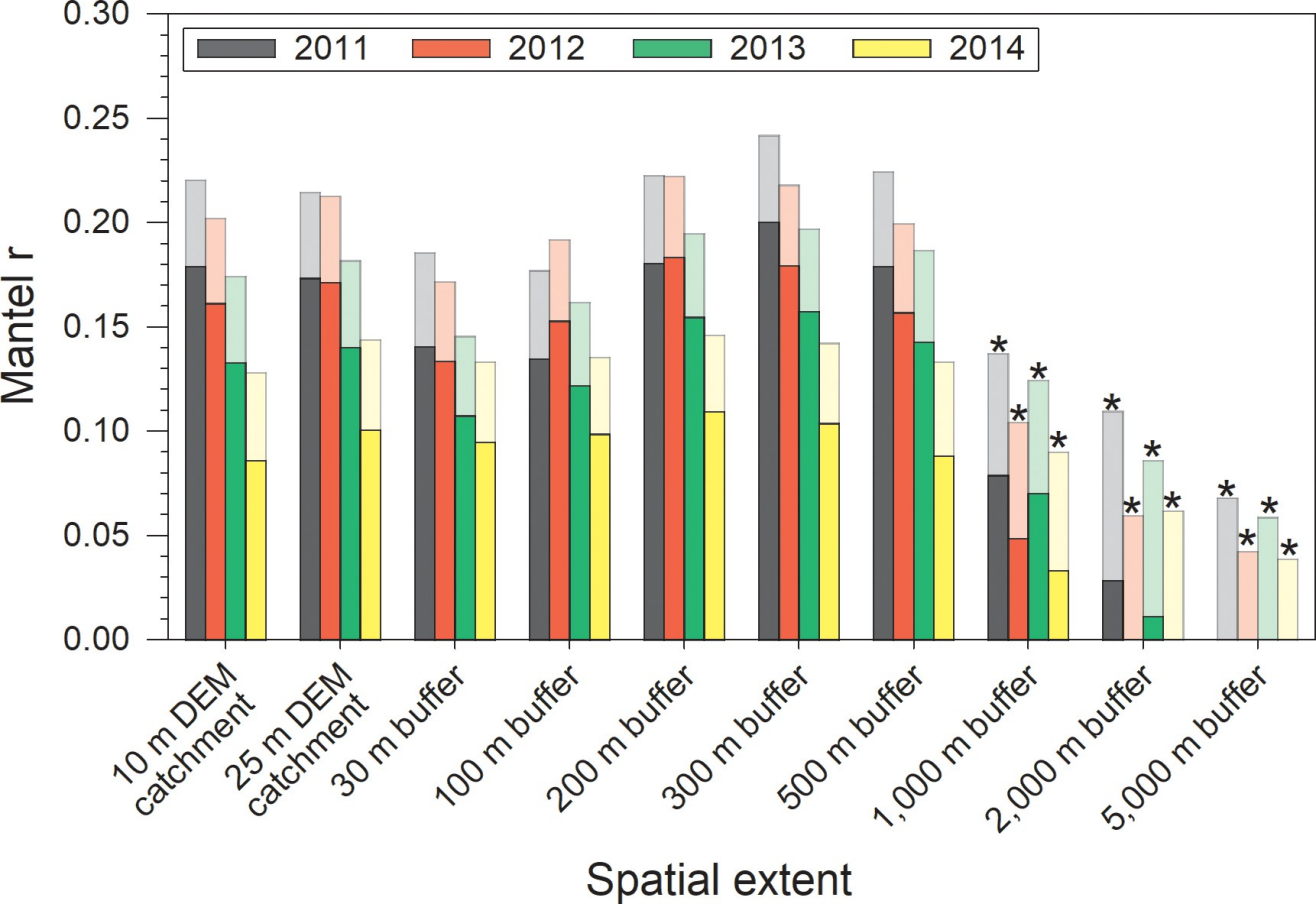
Comparison of simple (faded bars, background) and partial (darker bars, foreground) Mantel test results examining concordance of wetland physicochemical conditions and land cover for 48 marshes. The partial Mantel r_M_ values represent the remaining concordance between the physicochemical and land-cover data after linearly controlling for inter-wetland distances. The difference between faded and darker bars reflects the magnitude of concordance attributable to inter-wetland distances. Note that this difference comprises a large proportion of the concordance once buffers > 500 m in radius. Asterisks indicate partial Mantel *r*_M_ values that were not significantly different from 0, based on 90% confidence intervals (not shown for figure clarity).

In terms of physicochemical conditions, controlling for inter-wetland distances accounted for a substantial portion of the concordance that would otherwise have been attributed to land cover (Fig 6; S2 File). Consequently, land cover was strongly and significantly concordant with physicochemical conditions only when the landscape was confined to within 500 m of the wetland boundary, though *r*_M_ never exceeded 0.2. Land cover at or beyond the 1,000 m radius buffer exhibited only weak (*r*_M_ < 0.1) and non-significant concordance with physicochemical conditions, though inter-wetland distances (i.e. region) was strongly concordant with physicochemical conditions (Fig 6).

**Fig 7:**
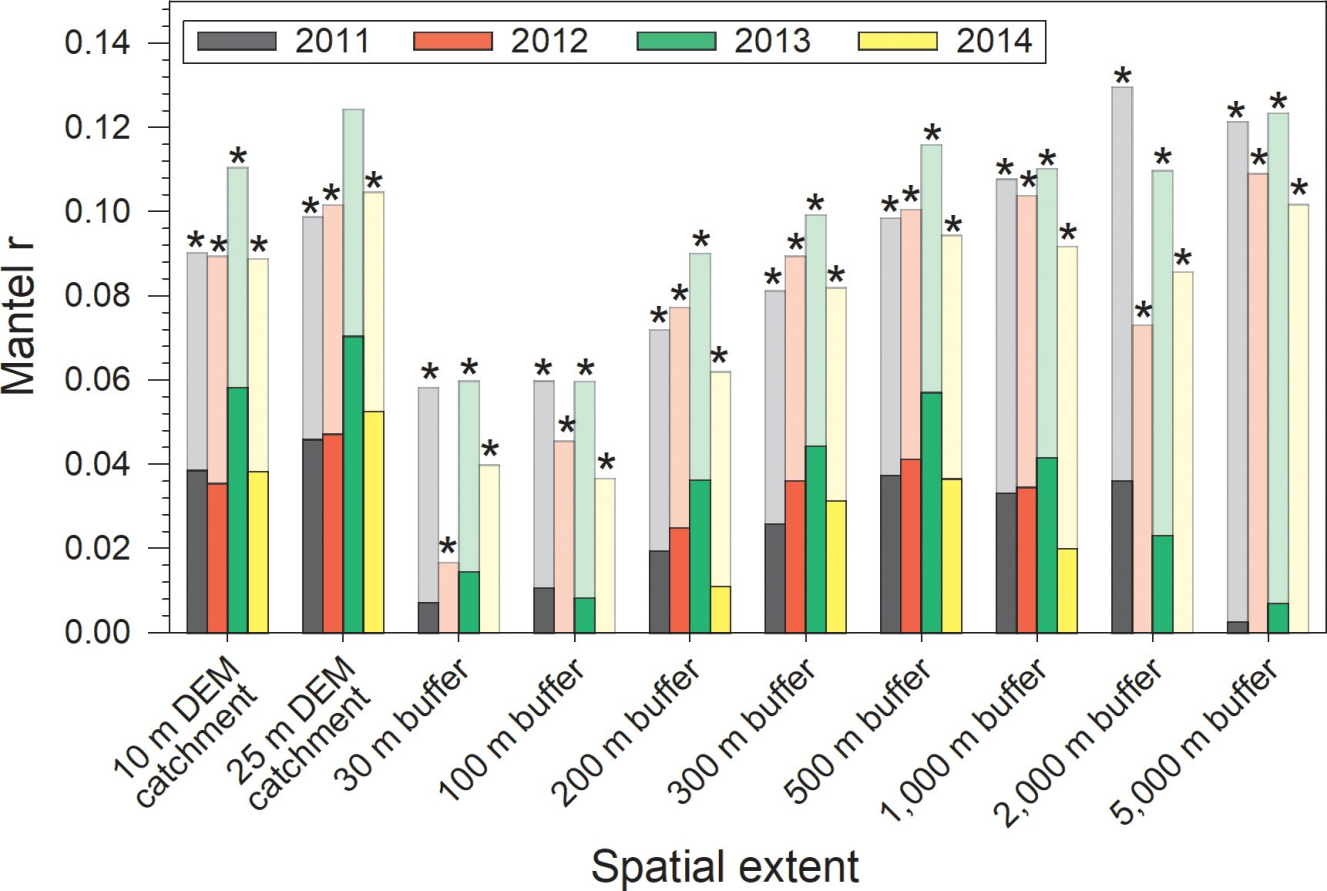
Comparison of simple (faded bars, background) and partial (darker bars, foreground) Mantel test results examining concordance of wetland vegetation community composition and land cover for 48 marshes. The partial Mantel *r*_M_ values represent the remaining concordance between the vegetation and land-cover data after linearly controlling for inter-wetland distances. The difference between faded and darker bars reflects the magnitude of concordance attributable to inter-wetland distances. Asterisks indicate partial Mantel *r*_M_ values that were not significantly different from 0, based on 90% confidence intervals (not shown for figure clarity).

Similarly, controlling for inter-wetland distances explained most of the concordance between the land cover dissimilarity matrices and the vegetation composition dissimilarity matrix. Only the comparison involving 2013 land cover from the 25 m DEM catchment was significantly related to vegetation conditions (*p* = 0.043; Fig 7; S3 File). Consequently, we conclude that any concordance between vegetation and surrounding land cover is negligible, though inter-wetland distance (i.e., region) is strongly concordant with vegetation dissimilarity.

Lastly, the wetland physicochemical and vegetation dissimilarity matrices were strongly and significantly concordant with each other, even after removing the influence of inter-wetland distance (partial Mantel *r*_M_ = 0.195, *p* = 0.007). Note this is one of the largest *r*_M_ values observed; more than double that of any of the *r*_M_ values from land cover vs. vegetation comparisons.

## Discussion

Our results suggest that the local landscape structure is having little direct influence on wetland vegetation community composition via processes related to propagule sources and dispersal, but rather that vegetation composition is dependent on wetland physicochemical conditions (which *are* affected by surrounding land cover) and on regional factors such as the vegetation species pool and geographic gradients in climate, soil type, and land use.

### Role of local landscapes

We were interested in exploring the scale of effect at which vegetation community composition and *in situ* physicochemical conditions in wetlands are sensitive to landscape and regional factors, and so we employed a nested buffer design to characterize the land cover composition around each wetland. Thus, our primary focus is the relative strength of concordance between dissimilarity in surrounding land cover from different landscape extents and dissimilarity in physicochemical conditions among wetlands.

We had expected that the vegetation community in a wetland would be influenced both by its physicochemical conditions [1, 56–57] and by the direct effects of land cover on species dispersal and propagule sources [5, 14–15]. We found that wetland vegetation was most concordant with land cover within 500 m of the wetland boundaries, though this concordance was weak (*r*_M_ < 0.05) and non-significant. This was surprising, as prior variance partitioning relating vegetation-based measures of wetland integrity to surrounding land cover in the Parkland natural region of Alberta found that even after factoring out the influence of inter-wetland distance, land cover within 500 m of wetland margins explained >40% of the variance in wetland integrity scores [23]. Yet, perhaps this should have been anticipated, as land-cover data in that study was able to explain >75% of the variance in wetland integrity scores when variations in location were not factored out. Notably, though not significantly different from 0, the *r*_*M*_ values among the buffers were highest at the 500 m buffer size, in agreement with the optimal buffer size identified by [23]. Because the reduction in concordance between land cover and vegetation was so large, especially at the largest buffer extents, we hypothesize that biogeographical factors have a comparatively strong influence on vegetation community composition in these wetlands. This is likely through differences in regional species pools and higher local immigration rates, which result in more distant wetlands possessing more dissimilar vegetation communities [15, 18, 56].

In contrast, physicochemical conditions were strongly (*r*_M_ ~ 0.1–0.2) and significantly concordant with land cover from within 200–500 m of the wetland boundary. In other words, we found strong evidence that surrounding land cover within 500 m influences wetland physicochemical conditions, but weak evidence to support a direct link between land cover and wetland vegetation, regardless of landscape extent. This contention is supported by previous research concluding that the relationship between land cover and wetland physicochemical conditions is limited in spatial extent [27, 56–59].

### Symmetrical buffers vs. hydrologic catchments

We hypothesized that wetland catchments reflecting the actual contributing area would show stronger concordance between land cover composition and physicochemical and vegetation variables than symmetrical buffers, as others have indicated e.g., [27–28]. We therefore contrasted the strength of concordance with land cover extracted from symmetrical buffers vs. land cover extracted from topographically delineated catchments. Interestingly, for both vegetation composition and physicochemical conditions, there was little material difference in the strength of concordance with land cover from catchments and land cover extracted from symmetrical buffers between 200 and 500 m in radius. While the catchments are representative of the hydrologically contributing area around the wetlands [24, 28], it is possible that surface runoff is not the major delivery vector of sediments, nutrients and contaminants to our prairie pothole wetlands. Due to naturally high infiltration rates in prairie soils and a semi-arid climate, little overland runoff is believed to reach prairie pothole wetlands during the active growing season [25]. Instead, most of the runoff occurs as snowmelt over frozen soil, when infiltration is low [25]. This may explain differences between our conclusions and those of [27] who were working in central Europe where the climate is less arid (mean annual precipitation of 840+ mm), resulting in more summertime runoff within wetland catchments and thus a stronger influence of catchment-scale land cover on water and sediment conditions. Similarly, the weak concordance we observed between local land cover and vegetation composition may be a consequence of minimal water-mediated propagule transport during the growing season, which can be a significant transportation vector for both upland and aquatic plants in regions with greater surface runoff [60]. Of course, snowmelt may be an important source of contaminants in agricultural landscapes e.g., [12], but processes not constrained by catchment boundaries, such as groundwater connections [60] or aerial deposition of sediments [8], may serve as important vectors influencing physicochemical conditions and vegetation in our prairie pothole wetlands. Regardless, our results indicate that the associations between land cover and wetland conditions are quite robust to spatial extent and less sensitive to runoff processes than anticipated. Though we are surprised by this conclusion, it does allay concerns around reliance on buffers in homogenous landscapes, or where high resolution DEMs or catchment polygons are not available.

### Role of *in situ* physicochemical conditions

To contrast the importance of local landscape condition with the importance of *in situ* physicochemical conditions on vegetation composition, we also measured the concordance between dissimilarity in wetland physicochemical conditions and dissimilarity in wetland vegetation communities directly. The strong (*r*_M_ = 0.195) and significant concordance between these dissimilarity matrices suggests that, as others have previously indicated [1, 58], wetland vegetation is more strongly influenced by *in situ* physicochemical conditions than by land cover in the local landscape (*r*_M_ < 0.1), regardless of landscape extent or delineation method. We cannot infer the causal direction of this strong association, but suggest it is most likely due to the direct influence of chemical and hydrologic conditions on the composition of vegetation e.g., [13], reflecting resource limitations and other environmental constraints of the capacity of plants to establish, compete and persist in their wetland habitat [15]. Yet, the physicochemical-vegetation association may also reflect the capacity of wetland vegetation to modify water and sediment conditions e.g., [58], or some unmeasured factor which controls both biotic and abiotic wetland conditions.

### Role of regional differences and inter-wetland distance

Our study wetlands span a latitudinal gradient that encompasses two natural regions with distinct climates, species pools, and land use patterns. For example, there is more forest cover in the Parkland vs. more native prairie in the Grassland; more cropping in the Parkland vs. more livestock grazing in the Grassland [31]. Thus, before we could explore patterns in the strength of concordance among the land cover, physicochemical and vegetation dissimilarity matrices, it was necessary to first factor out the influence of inter-wetland distances that more likely reflect geographic gradients in land cover and pertinent climate and soil characteristics or species distributions and dispersal rates [56]. Generally, we found strong and significant concordance between inter-wetland distances and both physicochemical and vegetation dissimilarity matrices. We suspect that this reflects the importance of regional differences in climate, soils or species pools [18]. This suggests that regional processes are determinants of *in situ* physicochemical conditions and vegetation community composition of importance equivalent to or greater than that of land cover in the local landscape.

### Geospatial data quality

We delineated wetland catchments using two topographic datasets of differing spatial grain (10 m and 25 m pixel DEMs), with the finer resolution DEM typically yielding smaller catchments (Table 1). Despite the difference in catchment size, we observed similarity in land cover composition between the two catchments. We attribute this to the relatively coarse resolution of the land-cover data (30 m pixels). The result of this spatial mismatch is that many of the same land cover pixels were included in both 10 m and 25 m catchments (e.g., Fig 1). The extremely high concordance between the land cover measured within the two catchment types supports this contention (concordance values averaged across the four years: mean *r*_M_ = 0.8720, mean *p* <0.0001).

The coarse resolution of the land-cover data (30 m pixels) is also of consequence when considering the smaller buffer sizes. We found that concordance between land cover and wetland condition was lower for the 30 m and 100 m buffers relative to 200 m and 500 m buffers, yet it is possible that low concordance in smaller buffers was a product of error in individual pixels’ classifications. Though the overall accuracy of the land cover data is 85% in our study area [45], the classifications of individual pixels may reflect greater error if they are mixed pixels [59]. Thus, it is essential to consider the data resolution when determining optimal buffer sizes.

For other applications involving delineated catchments (e.g., hydrologic modelling), previous work suggests that using high resolution DEMs (<10 m resolution) can improve fine-scale topographical detail [26], they typically do not produce catchments that considerably differ in size or shape from those delineated at moderate resolutions [28]. Furthermore, the acquisition of higher resolution topographic data for similar studies may not be a justifiable expense unless the geospatial data describing landscape features (e.g., land cover) are also represented at a very high spatial resolution [27].

## Conclusions

Our main contribution is in demonstrating that significant associations between surrounding land cover and physicochemical conditions in marshes of the Prairie Pothole Region are robust to the manner and spatial scale of land cover extraction and the vintage of land-cover data within a four-year window preceding field work. Concerns around access to catchment polygons or selection of an appropriate buffer size when relating land cover to physicochemical conditions or vegetation in marshes may be unnecessary, providing land-cover data is relatively recent (<5 years since collection) and proximate (≤500 m beyond wetland margins).

At spatial extents >500 m, we found that the observed association of physicochemical conditions with land cover was substantially reduced, which demonstrated a distance threshold of influence of land cover on physicochemical conditions in the wetlands. Once we accounted for inter-wetland distances, we detected only weak and mostly non-significant concordance between land cover and wetland vegetation composition, though vegetation remained strongly concordant with physicochemical conditions. We therefore conclude that land cover has little direct influence on vegetation composition, which is instead directly influenced by physicochemical conditions and inter-wetland distances that reflect species pool differences between natural regions and dispersal distances. Our results thus lend empirical support to prior warnings e.g., [9, 60] that in large study areas, spatial effects should be tested for and controlled to avoid drawing spurious conclusions on the nature of landscape-environment-biota relationships.

## Supporting information

S1 Table Summary statistics of land cover surrounding wetlands in the Grassland and Parkland. Summary of the land cover surrounding study wetlands in the Grassland (*n* = 24) and Parkland (*n* = 24) natural regions. Land cover values are expressed as percentage areal cover within a 300 m buffer around the wetland (the spatial extent where land cover was most concordant with both physicochemical conditions and vegetation communities).
(DOCX)

S2 Table Summary of physicochemical and hydrological measurements taken from study wetlands. Summary of the 45 water and soil analytes measured at the wetlands in 2014 used to construct the environmental dissimilarity matrix. Analytes are grouped by substrate (soil or water) and type (e.g., contaminants, ions, nutrients). Averages are presented for all 48 study wetlands combined and for the Grassland and Parkland natural regions separately (*n* = 24 each). All analytes were measured once during the study period except for *in situ* measures of turbidity, pond depth, dissolved oxygen, electrical conductivity, pH and temperature of water, which were measured once during each of five site visits and then averaged for analysis. To illustrate the variability in environmental conditions as non-natural land cover increases, sites are also binned according to the extent of cropland, developed land and pasture surrounding the site within a 500 m buffer in 2013: low disturbance (*n* = 22) represents sites with 0–25% non-natural cover; medium disturbance (*n* = 8) represents sites with 25–75% non-natural cover; and high disturbance (*n* = 18) represents sites with 75–100% non-natural cover.
(DOCX)

S1 File Principal components analysis and non-metric multidimensional scaling ordination results. Details on ordinations of physicochemical and vegetation data by PCA and NMS, respectively. Results are presented in two tables and two figures.
(DOCX)

S2 File Results of 40 Mantel and partial Mantel tests comparing land cover to wetland physicochemical conditions. Land cover was calculated as the percent cover of nine land cover types within ten landscape extents around the wetlands for each of four years. 90% confidence intervals (CIs) were calculated around the Mantel *r*_M_ values (coefficients indicating the level of similarity between two dissimilarity matrices), while partial Mantel test results represent the remaining land cover × physicochemical concordance after controlling for inter-wetland geographic distances. The significance of *r*_M_ values was determined at *α* = 0.05.
(DOCX)

S3 File Results of 40 Mantel and partial Mantel tests comparing land cover to wetland vegetation community composition. Land cover was calculated as the percent cover of nine land cover types within ten landscape extents around the wetlands for each of four years. 90% confidence intervals (CIs) were calculated around the Mantel *r*_M_ values (coefficients indicating the level of similarity between two dissimilarity matrices), while partial Mantel test results represent the remaining land cover × vegetation concordance after controlling for inter-wetland geographic distances. The significance of *r*_M_ values was determined at *α* **=** 0.05.
(DOCX)
