# Supplementary material for: Concordance in wetland physicochemical conditions, vegetation, and surrounding land cover is robust to data extraction approach
Source: PLoS One. 2019 May 31;14(5):e0216343. doi: 10.1371/journal.pone.0216343 (PMC6544339; doi:10.1371/journal.pone.0216343)
Supplement: S1 Table — Summary of the land cover surrounding study wetlands in the Grassland (n = 24) and Parkland (n = 24) natural regions. Land cover values are expressed as percentage areal cover within a 300 m buffer around the wetland (the spatial extent where land cover was most concordant with both physicochemical conditions and vegetation communities). (DOCX) [file pone.0216343.s001.docx]

S1 Table. Summary statistics of land cover surrounding wetlands in the Grassland and Parkland

Table S1. Summary of the land cover surrounding study wetlands in the Grassland (*n* = 24) and Parkland (*n* = 24) natural regions. Land cover values are expressed as percentage areal cover within a 300 m buffer around the wetland (the spatial extent where land cover was most concordant with both physicochemical conditions and vegetation communities).

| Land Cover Class | Region | Mean | Median | SD | Range |
| --- | --- | --- | --- | --- | --- |
| Cropland | Grassland | 24.38 | 1.75 | 32.51 | 0.00 - 88.15 |
|  | Parkland | 35.38 | 0.91 | 42.50 | 0.00 - 100.00 |
| Developed | Grassland | 3.94 | 2.57 | 6.13 | 0.00 - 28.51 |
|  | Parkland | 2.21 | 1.34 | 2.45 | 0.00 - 6.61 |
| Exposed | Grassland | 0.26 | 0.00 | 0.48 | 0.00 - 1.46 |
|  | Parkland | 0.17 | 0.00 | 0.50 | 0.00 - 2.36 |
| Forests | Grassland | 0.09 | 0.00 | 0.34 | 0.00 - 1.60 |
|  | Parkland | 20.21 | 1.86 | 29.95 | 0.00 - 80.86 |
| Grassland | Grassland | 54.22 | 69.65 | 42.10 | 0.00 - 100.00 |
|  | Parkland | 5.47 | 0.00 | 18.43 | 0.00 - 88.03 |
| Pasture | Grassland | 9.83 | 0.00 | 15.41 | 0.00 - 53.35 |
|  | Parkland | 17.06 | 3.90 | 25.02 | 0.00 - 74.67 |
| Shrubland | Grassland | 0.84 | 0.11 | 2.59 | 0.00 - 12.77 |
|  | Parkland | 13.73 | 8.76 | 18.96 | 0.00 - 86.63 |
| Water | Grassland | 0.39 | 0.00 | 0.73 | 0.00 - 2.93 |
|  | Parkland | 1.45 | 0.00 | 3.20 | 0.00 - 12.83 |
| Wetland | Grassland | 6.06 | 1.92 | 9.61 | 0.00 - 43.05 |
|  | Parkland | 4.33 | 2.82 | 4.79 | 0.00 - 14.66 |
