# Supplementary material for: Concordance in wetland physicochemical conditions, vegetation, and surrounding land cover is robust to data extraction approach
Source: PLoS One. 2019 May 31;14(5):e0216343. doi: 10.1371/journal.pone.0216343 (PMC6544339; doi:10.1371/journal.pone.0216343)
Supplement: S2 Table — Summary of the 45 water and soil analytes measured at the wetlands in 2014 used to construct the environmental dissimilarity matrix. Analytes are grouped by substrate (soil or water) and type (e.g., contaminants, ions, nutrients). Averages are presented for all 48 study wetlands combined and for the Grassland and Parkland natural regions separately (n = 24 each). All analytes were measured once during the study period except for in situ measures of turbidity, pond depth, dissolved oxygen, electrical conductivity, pH and temperature of water, which were measured once during each of five site visits and then averaged for analysis. To illustrate the variability in environmental conditions as non-natural land cover increases, sites are also binned according to the extent of cropland, developed land and pasture surrounding the site within a 500 m buffer in 2013: low disturbance (n = 22) represents sites with 0–25% non-natural cover; medium disturbance (n = 8) represents sites with 25–75% non-natural cover; and high disturbance (n = 18) represents sites with 75–100% non-natural cover. (DOCX) [file pone.0216343.s002.docx]

S2 Table. Summary of physicochemical and hydrological measurements taken from study wetlands

Table S2. Summary of the 45 water and soil analytes measured at the wetlands in 2014 used to construct the environmental dissimilarity matrix. Analytes are grouped by substrate (soil or water) and type (e.g., contaminants, ions, nutrients). Averages are presented for all 48 study wetlands combined and for the Grassland and Parkland natural regions separately (*n* = 24 each). All analytes were measured once during the study period except for *in situ* measures of turbidity, pond depth, dissolved oxygen, electrical conductivity, pH and temperature of water, which were measured once during each of five site visits and then averaged for analysis. To illustrate the variability in environmental conditions as non-natural land cover increases, sites are also binned according to the extent of cropland, developed land and pasture surrounding the site within a 500 m buffer in 2013: low disturbance (*n* = 22) represents sites with 0-25% non-natural cover; medium disturbance (*n* = 8) represents sites with 25-75% non-natural cover; and high disturbance (*n* = 18) represents sites with 75-100% non-natural cover.

| Type | Variable | Units | Average (Standard deviation) | Grassland Sites | Parkland Sites | Low Disturb. | Medium Disturb. | High Disturb. |
| --- | --- | --- | --- | --- | --- | --- | --- | --- |
| *Soil Analytes* |  |  |  |  |  |  |  |  |
| Contaminants | Total Detected Fungicides | count | 0.57 (0.78) | 0.51 | 0.63 | 0.32 | 0.50 | 0.90 |
| Contaminants | Total Detected Herbicides | count | 1.07 (1.10) | 1.18 | 0.97 | 0.74 | 1.00 | 1.52 |
| Contaminants | Total Detected Insecticides | count | 0.68 (0.98) | 0.93 | 0.43 | 0.56 | 1.13 | 0.63 |
| *In Situ* | Electrical Conductivity | mS/cm | 0.92 (1.20) | 0.80 | 1.05 | 0.86 | 1.20 | 0.88 |
| *In Situ* | pH | --- | 6.00 (0.89) | 5.71 | 6.29 | 5.86 | 6.28 | 6.05 |
| Ions | Calcium | mg/kg | 4160.61 (2990.02) | 2713.59 | 5607.64 | 4384.46 | 3924.47 | 3991.98 |
| Ions | Magnesium | mg/kg | 978.39 (722.82) | 731.61 | 1225.17 | 840.25 | 1349.57 | 982.25 |
| Ions | Potassium | mg/kg | 794.44 (324.26) | 941.50 | 647.38 | 783.79 | 824.22 | 794.23 |
| Ions | Sodium | mg/kg | 631.67 (1224.87) | 556.64 | 706.70 | 707.91 | 757.40 | 482.60 |
| Ions | Sodium Adsorption Ratio | --- | 12.74 (26.37) | 13.07 | 12.41 | 15.95 | 14.03 | 8.24 |
| Nutrients | Carbon:Nitrogen Ratio | --- | 12.50 (1.53) | 12.45 | 12.54 | 12.22 | 12.40 | 12.88 |
| Nutrients | Manganese | mg/kg | 57.79 (26.81) | 66.98 | 48.60 | 58.40 | 50.13 | 60.45 |
| Nutrients | Olsen Phosphorous | mg/kg | 58.56 (21.78) | 54.83 | 62.28 | 61.19 | 50.16 | 59.07 |
| Nutrients | Total Carbon | µg/L | 9.40 (7.41) | 5.67 | 13.12 | 10.86 | 8.57 | 7.98 |
| Nutrients | Total Nitrogen | µg/L | 0.77 (0.90) | 0.48 | 1.06 | 0.90 | 0.69 | 0.65 |
| Nutrients | Total Sulphur | mg/kg | 1929.04 (2292.94) | 1003.80 | 2854.28 | 2055.00 | 1845.40 | 1812.26 |
| Nutrients | Zinc | mg/kg | 9.68 (6.88) | 6.84 | 12.52 | 10.82 | 7.71 | 9.17 |
| Physical | Bulk Density | g/cm^3^ | 1.04 (0.24) | 1.20 | 0.88 | 1.03 | 1.00 | 1.08 |
| Physical | Gravimetric Water Content | % soil wet weight | 40.93 (12.78) | 33.03 | 48.83 | 41.35 | 40.29 | 40.70 |
| Physical | Loss-On-Ignition | % soil dry weight | 17.70 (12.87) | 11.50 | 23.90 | 20.44 | 16.33 | 14.97 |
| *Water Analytes* |  |  |  |  |  |  |  |  |
| Clarity | Total Suspended Solids | mg/L | 8.11 (15.31) | 4.15 | 12.08 | 7.32 | 10.69 | 7.94 |
| Clarity | Turbidity (*in situ* average) | NTU | 0.68 (0.47) | 0.64 | 0.73 | 0.59 | 0.97 | 0.67 |
| Contaminants | Total Detected Herbicides | count | 0.69 (1.29) | 0.79 | 0.58 | 0.14 | 0.63 | 1.39 |
| Contaminants | Total Detected Insecticides | count | 0.04 (0.20) | 0.00 | 0.08 | 0.00 | 0.00 | 0.11 |
| Hydrology | Amplitude:Maximum Depth Ratio | --- | 0.70 (0.34) | 0.77 | 0.63 | 0.77 | 0.68 | 0.62 |
| Hydrology | Maximum Pond Depth | m | 0.51 (0.24) | 0.50 | 0.52 | 0.45 | 0.52 | 0.58 |
| Hydrology | Pond Amplitude | m | 0.30 (0.15) | 0.34 | 0.26 | 0.31 | 0.23 | 0.33 |
| Hydrology | Pond Depth (average) | m | 0.36 (0.24) | 0.33 | 0.40 | 0.31 | 0.38 | 0.42 |
| Hydrology | Pond Dry Date | day of year | 297.42 (82.01) | 304.33 | 290.50 | 274.77 | 280.75 | 332.50 |
| *In Situ* | Dissolved Oxygen (average) | mg/L | 8.48 (2.80) | 9.29 | 7.67 | 8.98 | 8.19 | 7.99 |
| *In Situ* | Electrical Conductivity (average) | mS/cm | 2.56 (0.47) | 2.42 | 2.69 | 2.41 | 2.71 | 2.67 |
| *In Situ* | pH (average) | --- | 7.35 (0.81) | 7.49 | 7.21 | 7.44 | 7.18 | 7.32 |
| *In Situ* | Temperature (average) | °C | 18.21 (2.73) | 18.51 | 17.91 | 18.39 | 18.58 | 17.82 |
| Ions | Calcium | mg/L | 31.37 (38.01) | 21.36 | 41.38 | 22.56 | 26.26 | 44.42 |
| Ions | Chloride | mg/L | 5.46 (5.04) | 5.03 | 5.90 | 4.53 | 3.85 | 7.31 |
| Ions | Magnesium | mg/L | 23.73 (48.59) | 10.27 | 37.17 | 9.25 | 41.23 | 33.63 |
| Ions | Potassium | mg/L | 25.28 (13.95) | 22.78 | 27.79 | 19.05 | 26.02 | 32.58 |
| Ions | Sodium | mg/L | 61.46 (130.29) | 28.00 | 94.93 | 45.06 | 84.86 | 71.11 |
| Ions | Sodium Adsorption Ratio | --- | 11.46 (24.03) | 7.98 | 14.95 | 13.65 | 13.16 | 8.03 |
| Ions | Sulfate | mg/L | 161.17 (404.18) | 54.56 | 267.78 | 51.31 | 231.61 | 264.13 |
| Nutrients | Carbon:Nitrogen Ratio | --- | 11.54 (12.35) | 12.87 | 10.22 | 14.62 | 8.68 | 9.05 |
| Nutrients | Dissolved Organic Carbon | mg/L | 29.84 (11.81) | 27.90 | 31.78 | 30.62 | 33.21 | 27.38 |
| Nutrients | Total Carbon | µg/L | 2060.18 (2096.46) | 1686.44 | 2433.91 | 1949.95 | 2628.25 | 1942.42 |
| Nutrients | Total Nitrogen | µg/L | 251.52 (250.85) | 179.39 | 323.65 | 219.39 | 349.60 | 247.20 |
| Nutrients | Total Phosphorous | µg/L | 548.06 (867.46) | 596.79 | 499.33 | 588.14 | 344.50 | 589.56 |
