# Supplementary material for: Concordance in wetland physicochemical conditions, vegetation, and surrounding land cover is robust to data extraction approach
Source: PLoS One. 2019 May 31;14(5):e0216343. doi: 10.1371/journal.pone.0216343 (PMC6544339; doi:10.1371/journal.pone.0216343)
Supplement: S1 File — Details on ordinations of physicochemical and vegetation data by PCA and NMS, respectively. Results are presented in two tables and two figures. (DOCX) [file pone.0216343.s003.docx]

S1 File. Principal components analysis and non-metric multidimensional scaling ordination results

Details on ordinations of physicochemical and vegetation data by PCA and NMS, respectively. Results are presented in two tables and two figures.

For the 45 physicochemical variables included in analysis, any with a skewness value greater than 2 were log (x+1) transformed to improve multivariate normality. Then a PCA was run on a correlation cross-products matrix which yielded four significant axes that cumulatively explained 60% of the variance in the cross-products matrix: 26% on axis 1 (eigenvalue = 11.776, p = 0.001), 17% on axis 2 (eigenvalue = 7.776, p = 0.001), 10% on axis 3 (eigenvalue = 4.669, p = 0.001), and 7% on axis 4 (eigenvalue = 3.128, p = 0.001). Statistical significance of the axes was assessed by comparing the eigenvalue from the observed data with the range and mean eigenvalues from 999 randomizations of the dataset. The resulting scores for wetlands on each significant axis are presented in Table 1. The joint plot indicating the wetland’s relative positions along the first and second axis is presented in Figure 1. In this figure, vectors reflect the direction and magnitude of correlation between each physicochemical variable and the first two PCA axes for all variables with r^2^ > 0.200. Symbology indicates whether sites were in the Grassland (circle) or Parkland (square) Natural Region of Alberta.

We summarized variance in the vegetation community composition of the study wetlands with a NMS ordination.  First, any species present in fewer than four wetlands was excluded from analysis to control dataset sparsity, leaving 67 species.  Then, for each species, its percent cover was relativized by the maximum cover observed for that species to reduce dataset heterogeneity. The inter-wetland distances were then calculated using the Bray-Curtis dissimilarity measurement and the resulting matrix analysed.  Dimensionality was assessed by comparing 50 runs with real data to 50 runs with randomized data, using a maximum of 200 iterations to a stability criterion of 0.000001.  The optimal solution had 3 significant dimensions, with a final stress of 17.26 and instability < 0.00001 after 83 iterations.  This solution was rotated so that the greatest variance in the Bray-Curtis dissimilarity matrix was aligned along the first NMS axis. The first NMS axis explained 25.7%, the second 21.1%, and the third 18.5% of the variance, for a cumulative 65.3% explained by the ordination. The resulting scores for wetlands on each significant axis are presented in Table 2.  The joint plot indicating the wetland’s relative positions along the first and second axis is presented in Figure 2. In this figure, vectors reflect the direction and magnitude of correlation between each plant species and the first two NMS axes for all species with r^2^ > 0.150. Symbology indicates whether sites were in the Grassland (circle) or Parkland (square) Natural Region of Alberta.

Table 1. Wetland geographic coordinates and ordination scores on the first four axes from the principle components analysis of 45 physicochemical variables.

| Site | Region | Latitude | Longitude | PC1 | PC2 | PC3 | PC4 |
| --- | --- | --- | --- | --- | --- | --- | --- |
| 152 | Grassland | 50.36146 | 111.4234 | 3.0005 | 0.2899 | 1.271 | 1.4951 |
| 153 | Grassland | 50.51392 | 111.5009 | 2.072 | -0.5196 | 0.1049 | -0.4898 |
| 158 | Grassland | 50.55515 | 112.4954 | -0.4524 | -0.626 | -0.5786 | 2.2829 |
| 165 | Grassland | 50.31702 | 111.6562 | 4.2533 | -1.4254 | -1.2612 | 1.3712 |
| 173 | Grassland | 50.16439 | 111.5392 | 3.5002 | -1.3369 | 0.447 | -1.5614 |
| 202 | Grassland | 50.36546 | 112.0232 | 3.3954 | -0.4224 | -0.237 | 1.2617 |
| 203 | Grassland | 50.65699 | 112.4496 | 3.8562 | -0.4836 | -1.4036 | 0.0053 |
| 1001 | Grassland | 50.44743 | 111.89 | -1.6273 | -4.912 | 4.5758 | 2.4329 |
| 124 | Grassland | 51.31594 | 112.2355 | 1.5119 | -0.3916 | -2.8268 | -1.2599 |
| 131 | Grassland | 51.28776 | 112.2947 | 4.1207 | 1.5699 | 0.2289 | -0.486 |
| 133 | Grassland | 51.37127 | 112.1821 | 3.5197 | 2.1254 | 1.0451 | 0.2068 |
| 135 | Grassland | 51.49275 | 112.3819 | -1.3244 | -2.7376 | -4.0155 | 0.5933 |
| 142 | Grassland | 51.4136 | 112.1314 | 3.1222 | 3.3314 | -0.668 | -0.245 |
| 145 | Grassland | 51.60375 | 112.2063 | -4.7927 | -1.0689 | 0.2334 | -0.6047 |
| 149 | Grassland | 51.47505 | 112.0392 | -2.4425 | -1.987 | -1.7894 | -2.9457 |
| 184 | Grassland | 51.41711 | 112.5682 | 1.9471 | -1.252 | -3.3907 | -1.4131 |
| 98 | Grassland | 51.90163 | 111.6973 | 0.5646 | -5.6658 | 1.1009 | 2.7879 |
| 101 | Grassland | 51.03864 | 111.3166 | 3.1416 | 1.3732 | -0.0512 | -0.4052 |
| 109 | Grassland | 51.01003 | 111.8337 | 0.3135 | -3.9652 | -0.8895 | -1.5828 |
| 110 | Grassland | 51.53699 | 111.5053 | -0.5898 | -3.3483 | -2.3388 | 1.7931 |
| 115 | Grassland | 51.50547 | 111.2228 | 2.038 | -1.9722 | -3.0989 | -0.1288 |
| 117 | Grassland | 51.19809 | 111.5391 | 3.3005 | -0.4519 | -2.6016 | -1.2816 |
| 186 | Grassland | 51.83352 | 111.7223 | -0.1371 | -2.9315 | 0.601 | 2.0073 |
| 188 | Grassland | 51.52898 | 111.328 | 0.8182 | -2.7869 | 0.7026 | 2.1368 |
| 67 | Parkland | 52.4659 | 112.6971 | 3.3568 | 2.5157 | 1.5864 | 0.2031 |
| 89 | Parkland | 52.34653 | 112.9284 | -0.4046 | -1.5785 | -2.2304 | -2.3276 |
| 90 | Parkland | 52.34706 | 112.8723 | 0.7349 | 1.3964 | -1.9784 | -0.9539 |
| 194 | Parkland | 52.2196 | 113.4426 | -4.5216 | 0.8688 | -1.849 | -0.4439 |
| 195 | Parkland | 52.41014 | 113.044 | -3.3481 | 0.7845 | 1.5798 | 3.1974 |
| 1002 | Parkland | 52.50928 | 113.2244 | -0.5234 | 8.8752 | 2.7597 | 0.2677 |
| 1003 | Parkland | 51.88395 | 112.6318 | -1.4968 | -0.0323 | -0.6184 | -0.2574 |
| 1004 | Parkland | 52.18619 | 113.0199 | -5.1169 | -3.8026 | 3.5345 | 1.0198 |
| 31 | Parkland | 52.73904 | 113.3523 | -3.421 | 3.8245 | -0.6884 | 3.7083 |
| 32 | Parkland | 52.59304 | 113.5987 | -0.3618 | 2.0554 | -4.7435 | 3.8832 |
| 35 | Parkland | 53.07182 | 113.4282 | 4.3706 | 1.6673 | 4.3042 | -0.9729 |
| 56 | Parkland | 52.94941 | 112.6346 | 2.2887 | -1.919 | 3.1872 | -2.7061 |
| 190 | Parkland | 53.0911 | 113.1969 | -4.4772 | 1.8054 | 1.8147 | -3.62 |
| 1005 | Parkland | 52.92774 | 114.1974 | 1.2733 | 5.6182 | 0.4502 | 0.0672 |
| 1006 | Parkland | 52.5575 | 113.6309 | -3.7012 | 5.3223 | -2.4382 | 1.3927 |
| 1007 | Parkland | 53.23402 | 112.8747 | -5.4422 | 4.0518 | -1.6779 | -1.9579 |
| 10 | Parkland | 52.51476 | 112.648 | -7.5083 | 0.0624 | -0.1877 | -0.4209 |
| 13 | Parkland | 52.33939 | 112.2282 | 1.3219 | 1.0605 | 3.3728 | 1.4508 |
| 18 | Parkland | 52.58662 | 112.208 | 0.9151 | 2.0778 | 2.287 | 0.6463 |
| 25 | Parkland | 52.14865 | 111.8232 | 0.7464 | -0.0744 | 0.9401 | -1.4252 |
| 30 | Parkland | 52.38929 | 111.8738 | 3.8419 | -1.7997 | 1.3891 | -1.9849 |
| 182 | Parkland | 52.7306 | 112.4103 | 3.6679 | -0.7494 | 1.1339 | -1.702 |
| 187 | Parkland | 52.62298 | 112.6321 | -7.5717 | -0.9766 | 0.7975 | -1.2244 |
| 200 | Parkland | 52.47806 | 112.6138 | -7.7319 | -1.4583 | 2.1151 | -1.8098 |

**
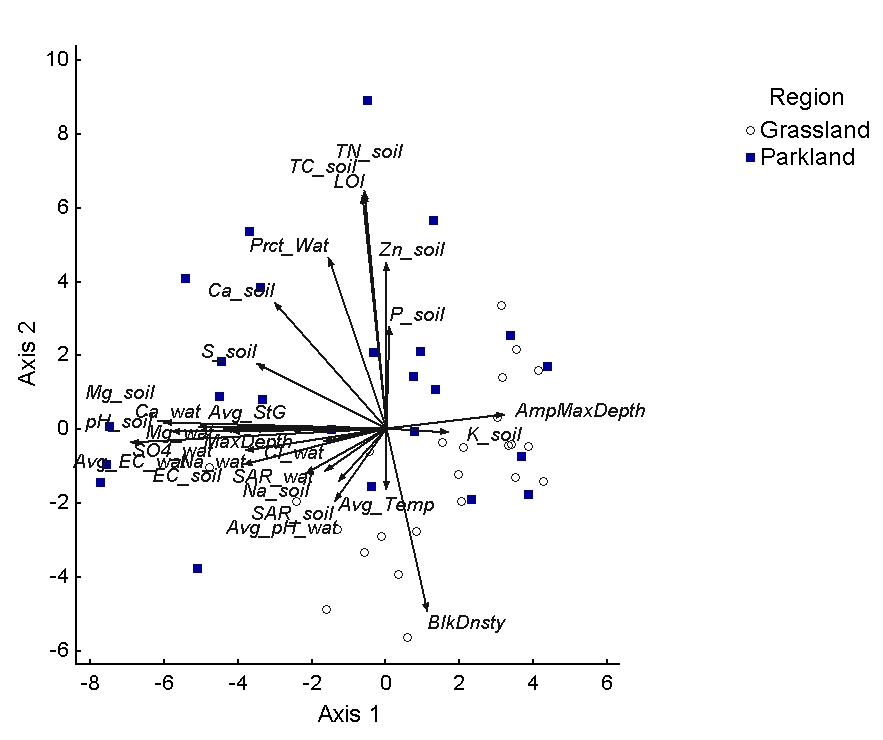
**

Figure 2. PCA ordination joint plot representing physicochemical variables overlain on wetland relative positions.

Table 2. Wetland geographic coordinates and ordination scores on the three significant axes from the NMS ordination of 67 plant species.

| Site | Region | Latitude | Longitude | NMS1 | NMS2 | NMS3 |
| --- | --- | --- | --- | --- | --- | --- |
| 152 | Grassland | 50.36146 | 111.4234 | 0.7867 | 0.2892 | -0.569 |
| 153 | Grassland | 50.51392 | 111.5009 | 0.167 | 0.4844 | -0.3837 |
| 158 | Grassland | 50.55515 | 112.4954 | 0.4084 | -0.0615 | -0.0668 |
| 165 | Grassland | 50.31702 | 111.6562 | 0.9885 | 0.3517 | 0.0662 |
| 173 | Grassland | 50.16439 | 111.5392 | 1.0535 | 0.0323 | -0.1055 |
| 202 | Grassland | 50.36546 | 112.0232 | 0.9162 | -0.0258 | -0.2282 |
| 203 | Grassland | 50.65699 | 112.4496 | 1.0608 | -0.6176 | 0.4633 |
| 1001 | Grassland | 50.44743 | 111.89 | 0.3391 | 0.8728 | -0.9253 |
| 124 | Grassland | 51.31594 | 112.2355 | 0.2711 | -0.2347 | 0.8135 |
| 131 | Grassland | 51.28776 | 112.2947 | 0.4782 | -0.0878 | 0.416 |
| 133 | Grassland | 51.37127 | 112.1821 | 0.5417 | -0.7976 | -0.5541 |
| 135 | Grassland | 51.49275 | 112.3819 | 0.193 | 0.2672 | 0.9111 |
| 142 | Grassland | 51.4136 | 112.1314 | -0.5233 | -0.2535 | -0.1114 |
| 145 | Grassland | 51.60375 | 112.2063 | 0.0089 | 0.0132 | 0.7453 |
| 149 | Grassland | 51.47505 | 112.0392 | -0.4381 | 0.4171 | -0.7788 |
| 184 | Grassland | 51.41711 | 112.5682 | 0.2895 | -0.2124 | -0.9601 |
| 98 | Grassland | 51.90163 | 111.6973 | 1.0182 | 0.1418 | 0.5126 |
| 101 | Grassland | 51.03864 | 111.3166 | 0.2657 | 0.114 | 0.1884 |
| 109 | Grassland | 51.01003 | 111.8337 | 0.3723 | -0.425 | -0.9779 |
| 110 | Grassland | 51.53699 | 111.5053 | 0.3535 | 0.1037 | 0.4602 |
| 115 | Grassland | 51.50547 | 111.2228 | 0.6392 | -0.0522 | -0.3918 |
| 117 | Grassland | 51.19809 | 111.5391 | 0.3448 | -0.322 | -0.0139 |
| 186 | Grassland | 51.83352 | 111.7223 | 0.4815 | 0.2108 | 0.6499 |
| 188 | Grassland | 51.52898 | 111.328 | 0.9763 | 0.5932 | -0.0335 |
| 67 | Parkland | 52.4659 | 112.6971 | -0.4427 | -0.5558 | -0.4145 |
| 89 | Parkland | 52.34653 | 112.9284 | -0.8971 | -0.2082 | -1.1233 |
| 90 | Parkland | 52.34706 | 112.8723 | -0.6123 | -0.0278 | 0.3973 |
| 194 | Parkland | 52.2196 | 113.4426 | -0.7736 | 0.7462 | 0.7876 |
| 195 | Parkland | 52.41014 | 113.044 | -0.8027 | 0.8088 | 0.2805 |
| 1002 | Parkland | 52.50928 | 113.2244 | -0.8029 | -0.6243 | 0.7111 |
| 1003 | Parkland | 51.88395 | 112.6318 | -1.1064 | -0.1633 | -0.3292 |
| 1004 | Parkland | 52.18619 | 113.0199 | 0.6199 | 0.7152 | 0.277 |
| 31 | Parkland | 52.73904 | 113.3523 | -0.2284 | 0.4346 | 0.5073 |
| 32 | Parkland | 52.59304 | 113.5987 | -0.7855 | 0.0817 | 0.1361 |
| 35 | Parkland | 53.07182 | 113.4282 | -0.8438 | 1.2013 | -0.8613 |
| 56 | Parkland | 52.94941 | 112.6346 | -0.2546 | -0.9378 | -0.7163 |
| 190 | Parkland | 53.0911 | 113.1969 | -0.6895 | 0.3881 | -0.3386 |
| 1005 | Parkland | 52.92774 | 114.1974 | -1.3298 | -0.6493 | 0.4973 |
| 1006 | Parkland | 52.5575 | 113.6309 | -0.9799 | -0.3148 | 0.222 |
| 1007 | Parkland | 53.23402 | 112.8747 | -0.7365 | 0.5899 | -0.1594 |
| 10 | Parkland | 52.51476 | 112.648 | -0.0597 | 0.3582 | 0.2033 |
| 13 | Parkland | 52.33939 | 112.2282 | -0.0106 | -0.3999 | 0.2276 |
| 18 | Parkland | 52.58662 | 112.208 | -0.5594 | -0.5438 | 0.5763 |
| 25 | Parkland | 52.14865 | 111.8232 | -0.0369 | -0.3471 | -0.014 |
| 30 | Parkland | 52.38929 | 111.8738 | -0.079 | -1.7741 | -0.0577 |
| 182 | Parkland | 52.7306 | 112.4103 | 0.577 | -0.8602 | -0.1496 |
| 187 | Parkland | 52.62298 | 112.6321 | -0.0962 | 0.5356 | 0.2407 |
| 200 | Parkland | 52.47806 | 112.6138 | -0.0622 | 0.7452 | -0.0266 |


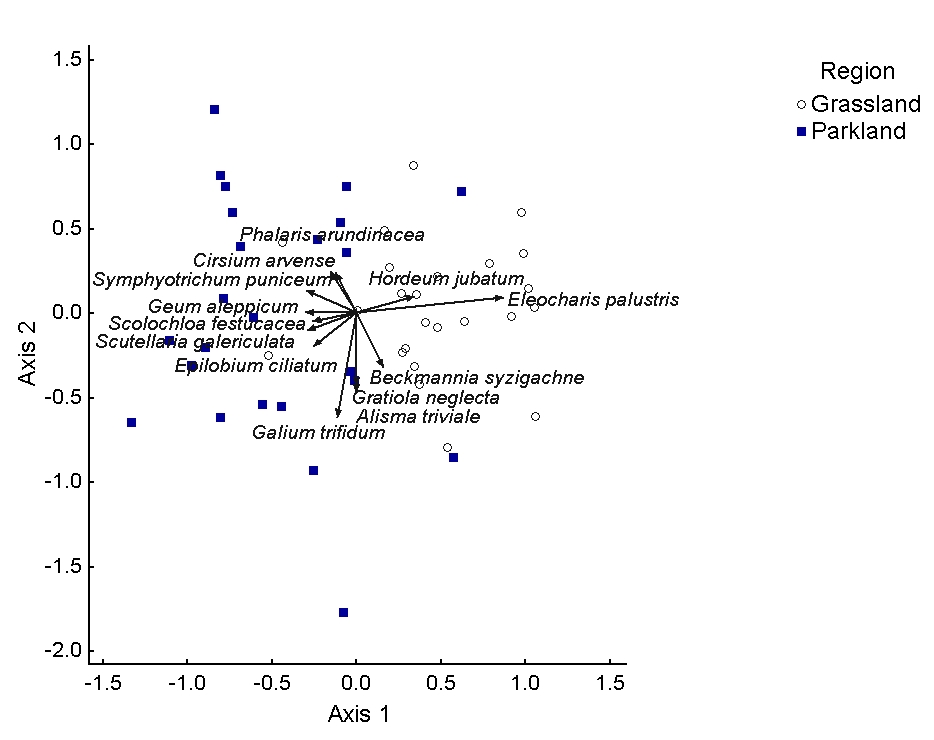


Figure 2. NMS ordination joint plot representing vegetation species overlain on wetland relative positions.
