# Supplementary material for: Concordance in wetland physicochemical conditions, vegetation, and surrounding land cover is robust to data extraction approach
Source: PLoS One. 2019 May 31;14(5):e0216343. doi: 10.1371/journal.pone.0216343 (PMC6544339; doi:10.1371/journal.pone.0216343)
Supplement: S3 File — Land cover was calculated as the percent cover of nine land cover types within ten landscape extents around the wetlands for each of four years. 90% confidence intervals (CIs) were calculated around the Mantel rM values (coefficients indicating the level of similarity between two dissimilarity matrices), while partial Mantel test results represent the remaining land cover × vegetation concordance after controlling for inter-wetland geographic distances. The significance of rM values was determined at α = 0.05. (DOCX) [file pone.0216343.s005.docx]

S3 File. Results of 40 Mantel and partial Mantel tests comparing land cover to wetland vegetation community composition

Table 1. Land cover was calculated as the percent cover of nine land cover types within ten landscape extents around the wetlands for each of four years. 90% confidence intervals (CIs) were calculated around the Mantel *r*_M_ values (coefficients indicating the level of similarity between two dissimilarity matrices), while partial Mantel test results represent the remaining land cover × vegetation concordance after controlling for inter-wetland geographic distances. The significance of *r*_M_ values was determined at *α* = 0.05.

|  |  | *Mantel Test* | | | | |  | *Partial Mantel Test* | |  |  |
| --- | --- | --- | --- | --- | --- | --- | --- | --- | --- | --- | --- |
| Spatial Extent | Year | *r*_M_ | Lower CI | Upper CI | CI width | *p*-value |  | *r*_M_ | *p*-value |  | *r*_M_ Change ^a^ |
| 10 m DEM Catchment | 2011 | 0.0904 | 0.0185 | 0.1671 | 0.1487 | 0.0172 |  | 0.0387 | 0.1777 |  | -0.0517 |
|  | 2012 | 0.0895 | 0.0177 | 0.1679 | 0.1502 | 0.0170 |  | 0.0356 | 0.1907 |  | -0.0539 |
|  | 2013 | 0.1105 | 0.0376 | 0.1915 | 0.1539 | 0.0040 |  | 0.0583 | 0.0652 |  | -0.0522 |
|  | 2014 | 0.0889 | 0.0227 | 0.1657 | 0.1429 | 0.0140 |  | 0.0384 | 0.1528 |  | -0.0505 |
| 25 m DEM Catchment | 2011 | 0.0989 | 0.0220 | 0.1800 | 0.1579 | 0.0097 |  | 0.0461 | 0.1284 |  | -0.0528 |
|  | 2012 | 0.1016 | 0.0275 | 0.1865 | 0.1590 | 0.0101 |  | 0.0473 | 0.1376 |  | -0.0543 |
|  | 2013 | 0.1244 | 0.0511 | 0.2055 | 0.1544 | 0.0022 |  | 0.0705 | 0.0433 |  | -0.0539 |
|  | 2014 | 0.1047 | 0.0347 | 0.1834 | 0.1487 | 0.0057 |  | 0.0527 | 0.0872 |  | -0.0521 |
| 30 m Buffer | 2011 | 0.0584 | -0.0102 | 0.1364 | 0.1465 | 0.0870 |  | 0.0074 | 0.4178 |  | -0.0510 |
|  | 2012 | 0.0169 | -0.0431 | 0.0834 | 0.1264 | 0.3329 |  | -0.0299 | 0.7361 |  | -0.0468 |
|  | 2013 | 0.0599 | -0.0116 | 0.1399 | 0.1515 | 0.0806 |  | 0.0147 | 0.3403 |  | -0.0452 |
|  | 2014 | 0.0400 | -0.0332 | 0.1196 | 0.1528 | 0.1530 |  | -0.0029 | 0.5127 |  | -0.0429 |
| 100 m Buffer | 2011 | 0.0599 | -0.0096 | 0.1395 | 0.1491 | 0.0762 |  | 0.0108 | 0.3787 |  | -0.0491 |
|  | 2012 | 0.0457 | -0.0171 | 0.1165 | 0.1337 | 0.1322 |  | -0.0058 | 0.5396 |  | -0.0515 |
|  | 2013 | 0.0597 | -0.0110 | 0.1390 | 0.1500 | 0.0718 |  | 0.0084 | 0.3986 |  | -0.0513 |
|  | 2014 | 0.0369 | -0.0297 | 0.1150 | 0.1447 | 0.1654 |  | -0.0112 | 0.5868 |  | -0.0480 |
| 200 m Buffer | 2011 | 0.0721 | -0.0002 | 0.1552 | 0.1553 | 0.0409 |  | 0.0196 | 0.3087 |  | -0.0525 |
|  | 2012 | 0.0774 | 0.0119 | 0.1546 | 0.1427 | 0.0311 |  | 0.0250 | 0.2792 |  | -0.0524 |
|  | 2013 | 0.0902 | 0.0178 | 0.1715 | 0.1537 | 0.0170 |  | 0.0365 | 0.1808 |  | -0.0537 |
|  | 2014 | 0.0621 | -0.0078 | 0.1390 | 0.1468 | 0.0668 |  | 0.0112 | 0.3810 |  | -0.0509 |
| 300 m Buffer | 2011 | 0.0813 | 0.0073 | 0.1600 | 0.1527 | 0.0274 |  | 0.0260 | 0.2643 |  | -0.0553 |
|  | 2012 | 0.0896 | 0.0204 | 0.1686 | 0.1482 | 0.0197 |  | 0.0363 | 0.1936 |  | -0.0532 |
|  | 2013 | 0.0994 | 0.0235 | 0.1808 | 0.1573 | 0.0078 |  | 0.0445 | 0.1334 |  | -0.0548 |
|  | 2014 | 0.0821 | 0.0177 | 0.1561 | 0.1384 | 0.0209 |  | 0.0315 | 0.2078 |  | -0.0506 |
| 500 m Buffer | 2011 | 0.0985 | 0.0265 | 0.1781 | 0.1516 | 0.0093 |  | 0.0376 | 0.1852 |  | -0.0610 |
|  | 2012 | 0.1006 | 0.0311 | 0.1794 | 0.1483 | 0.0059 |  | 0.0414 | 0.1455 |  | -0.0592 |
|  | 2013 | 0.1160 | 0.0419 | 0.1956 | 0.1538 | 0.0037 |  | 0.0571 | 0.0782 |  | -0.0588 |
|  | 2014 | 0.0945 | 0.0263 | 0.1678 | 0.1415 | 0.0127 |  | 0.0367 | 0.1634 |  | -0.0578 |
| 1,000 m Buffer | 2011 | 0.1078 | 0.0310 | 0.1869 | 0.1560 | 0.0055 |  | 0.0334 | 0.2273 |  | -0.0745 |
|  | 2012 | 0.1039 | 0.0335 | 0.1774 | 0.1439 | 0.0046 |  | 0.0347 | 0.1851 |  | -0.0692 |
|  | 2013 | 0.1102 | 0.0383 | 0.1872 | 0.1489 | 0.0043 |  | 0.0417 | 0.1595 |  | -0.0685 |
|  | 2014 | 0.0918 | 0.0220 | 0.1674 | 0.1455 | 0.0168 |  | 0.0202 | 0.2935 |  | -0.0716 |
| 2,000 m Buffer | 2011 | 0.1296 | 0.0520 | 0.2116 | 0.1595 | 0.0010 |  | 0.0363 | 0.2302 |  | -0.0933 |
|  | 2012 | 0.0732 | 0.0057 | 0.1458 | 0.1401 | 0.0419 |  | -0.0200 | 0.6547 |  | -0.0932 |
|  | 2013 | 0.1098 | 0.0353 | 0.1878 | 0.1526 | 0.0071 |  | 0.0233 | 0.2997 |  | -0.0865 |
|  | 2014 | 0.0858 | 0.0117 | 0.1624 | 0.1506 | 0.0303 |  | -0.0105 | 0.5734 |  | -0.0962 |
| 5,000 m Buffer | 2011 | 0.1214 | 0.0472 | 0.1980 | 0.1507 | 0.0031 |  | 0.0029 | 0.4778 |  | -0.1186 |
|  | 2012 | 0.1091 | 0.0405 | 0.1796 | 0.1391 | 0.0098 |  | -0.0137 | 0.6032 |  | -0.1228 |
|  | 2013 | 0.1234 | 0.0550 | 0.1940 | 0.1391 | 0.0048 |  | 0.0073 | 0.4551 |  | -0.1161 |
|  | 2014 | 0.1017 | 0.0300 | 0.1765 | 0.1466 | 0.0185 |  | -0.0239 | 0.6669 |  | -0.1257 |

^a^ *r*_M_ Change = change in *r*_M_ after controlling for geographic position (partial Mantel *r*_M_ – Mantel *r*_M_).


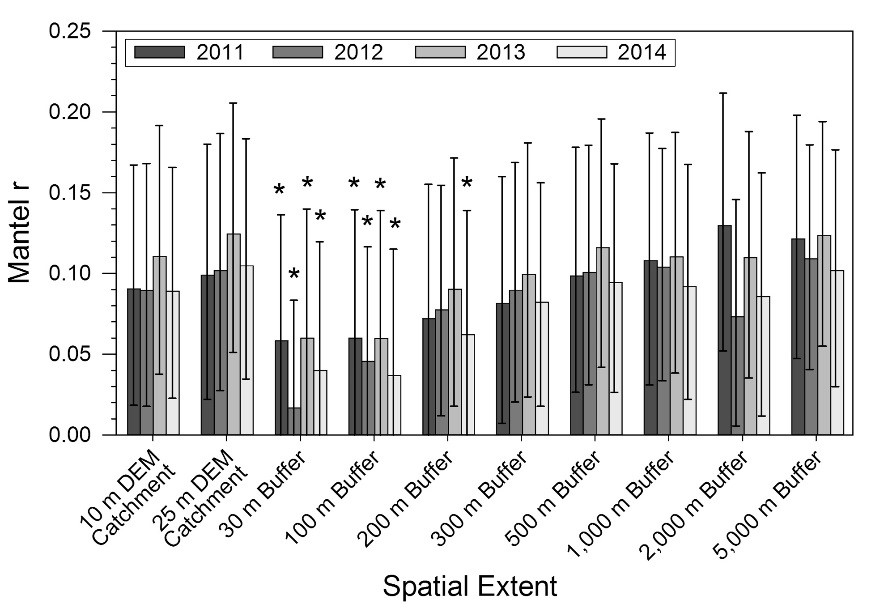


Figure 1. Mantel correlation coefficients from simple Mantel tests carried out comparing dissimilarity in land cover composition with dissimilarity in vegetation community composition in the 48 study wetlands without controlling for inter-wetland geographic distances. Confidence intervals are 90% CIs derived from bootstrapping (70%, without replacement). Asterisks indicate coefficients with confidence intervals that encompass 0. Lower case letters indicate non-overlapping confidence intervals.
